# Template and Temperature-Controlled Polymorph Formation in Squaraine Thin Films

**DOI:** 10.1021/acs.langmuir.2c01023

**Published:** 2022-07-20

**Authors:** Frank Balzer, Tobias Breuer, Gregor Witte, Manuela Schiek

**Affiliations:** † SDU Centre for Photonics Engineering, University of Southern Denmark, Sønderborg DK-6400, Denmark; ‡ Department of Physics, Philipps University of Marburg, Marburg D-35032, Germany; § Institute of Physics, University of Oldenburg, Oldenburg D-26111, Germany; ∥ Center for Surface- and Nanoanalytics (ZONA), Institute for Physical Chemistry (IPC) & Linz Institute for Organic Solar Cells (LIOS), Johannes Kepler University, Linz A-4040, Austria

## Abstract

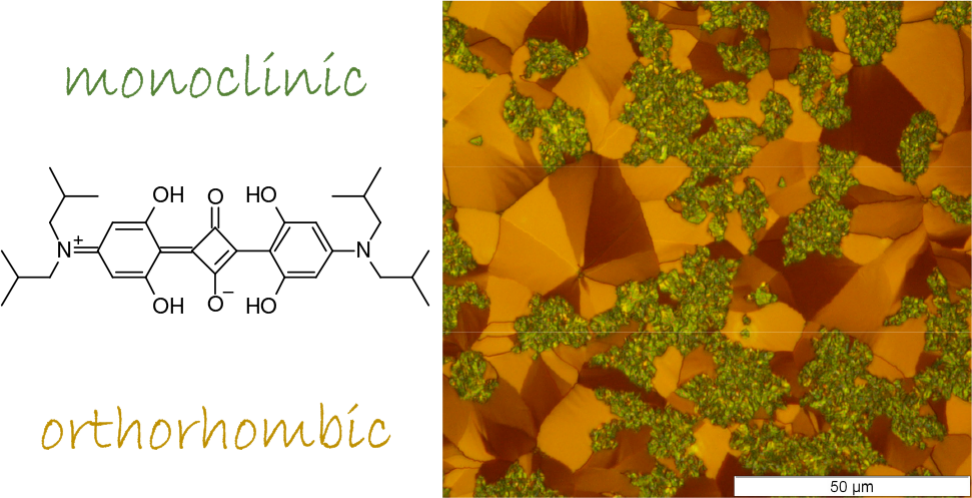

Controlling the polymorph formation in organic semiconductor thin films by the choice of processing parameters is a key factor for targeted device performance. Small molecular semiconductors such as the prototypical anilino squaraine compound with branched butyl chains as terminal functionalization (SQIB) allow both solution and vapor phase deposition methods. SQIB has been considered for various photovoltaic applications mainly as amorphous isotropic thin films due to its broad absorption within the visible to deep-red spectral range. The two known crystalline polymorphs adopting a monoclinic and orthorhombic crystal phase show characteristic Frenkel excitonic spectral signatures of overall H-type and J-type aggregates, respectively, with additional pronounced Davydov splitting. This gives a recognizable polarized optical response of crystalline thin films suitable for identification of the polymorphs. Both phases emerge with a strongly preferred out-of-plane and rather random in-plane orientation in spin-casted thin films depending on subsequent thermal annealing. By contrast, upon vapor deposition on dielectric and conductive substrates, such as silicon dioxide, potassium chloride, graphene, and gold, the polymorph expression depends basically on the choice of growth substrate. The same pronounced out-of-plane orientation is adopted in all crystalline cases, but with a surface templated in-plane alignment in case of crystalline substrates. Strikingly, the amorphous isotropic thin films obtained by vapor deposition cannot be crystallized by thermal postannealing, which is a key feature for the spin-casted thin films, here monitored by polarized in situ microscopy. Combining X-ray diffraction, atomic force microscopy, ellipsometry, and polarized spectro-microscopy, we identify the processing-dependent evolution of the crystal phases, correlating morphology and molecular orientations within the textured SQIB films.

## 1 Introduction

**Figure 1:**
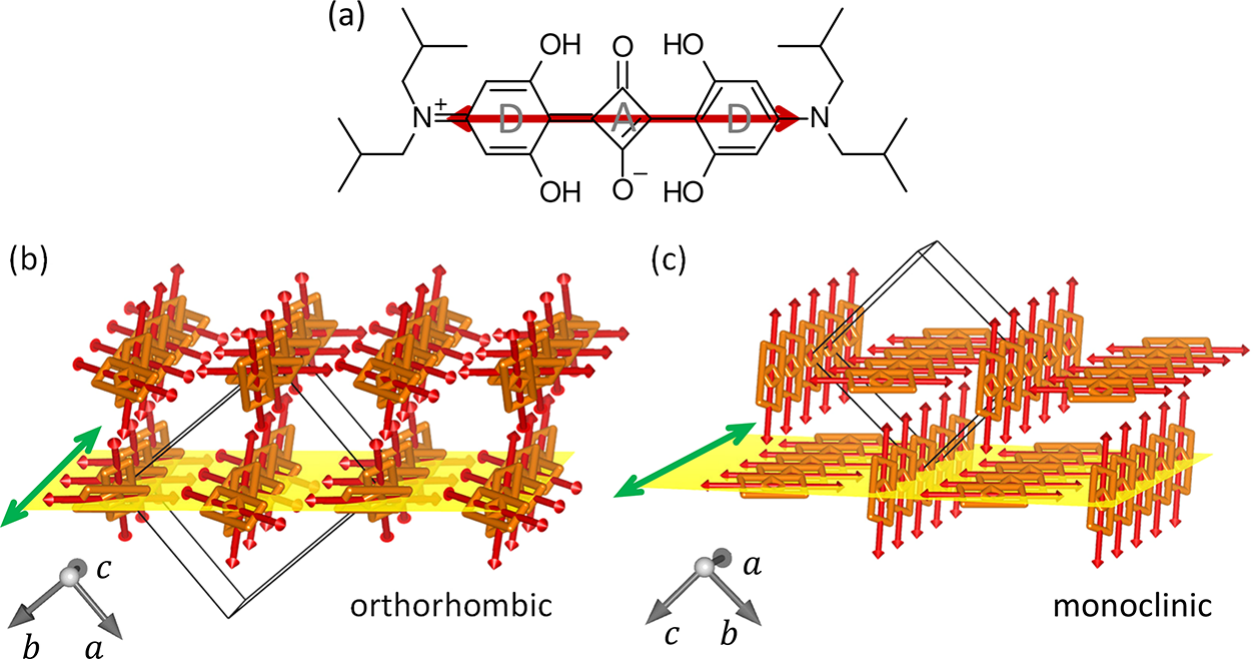
(a) Structural formula of the donor–acceptor–donor-type (D–A–D) SQIB molecule. The red arrow denotes the long molecular axis and the direction of the transition dipole moment for the S_0_ → S_1_ transition. Packing and stacking of SQIB molecules and the observed planes parallel to the surface for the two known polymorphs are depicted in (b) and (c) using VESTA.^32^ The primitive unit cells are denoted by thin black lines. The long molecular axes of the SQIB molecules, and with that the transition dipole moments, are shown by red arrows. Molecular backbones are depicted by orange rectangles to visualize their stacking. The molecular stacking directions are indicated by green arrows pointing parallel to the crystallographic *c*-axis and *a*-axis in (b) and (c), respectively. The yellow planes denote the (110) plane of the orthorhombic *Pbcn* phase (*Z* = 4) (b) and the (011) plane of the monoclinic *P*2_1_/*c* phase (*Z* = 2) (c), respectively.

Crystalline organic thin films often exhibit linear dichroism and birefringence.^1^ By structural design of the molecular building blocks, advanced functionality can be introduced including nonlinear optical properties^2^ or circular dichroism.^3,4^ In addition, fine-tuning can be obtained by selecting a specific polymorph through the processing conditions.^5−9^ The interest in polymorph selective growth of organic molecules is widespread, because the polymorph choice can determine the color of pigments and aggregates,^10,11^ improve optoelectronic device performance,^12−15^ or decide about the biofunctionality of, for instance, active drug ingredients.^16−19^ Even for monolayer J-aggregates, polymorphism determines the polarized light fluorescence.^20^ The dihydroxy anilino squaraine SQIB (2,4-bis[4-(*N*,*N*-diisobutylamino)-2,6-dihydroxyphenyl]squaraine), as sketched in Figure 1a, is a prototypical quadrupolar donor–acceptor–donor-type semiconductor compound. Because of the strong light matter interaction in the visible to deep-red region, squaraines have been widely used for various photovoltaic applications including xerography,^21,22^ solar cells,^23−28^ photodetectors,^29^ and neurostimulating photocapacitors.^30,31^

In case of SQIB, two polymorphs are known, each having multiple molecules in the primitive unit cell:^33,34^ a monoclinic *P*2_1_/*c* phase (*Z* = 2; CCDC code 1567209) and an orthorhombic *Pbcn* phase (*Z* = 4; CCDC code 1567104), as depicted in Figure 1b and c.

The optical properties of the condensed phases are dominated by Coulombic coupling between the molecular compounds. In a simplified picture according to the Kasha model, the excitonic properties can be described by linear combinations of multiple transition dipole moments.^37−41^ Here, the transition dipole moment is along the molecular backbone as depicted by red arrows in Figure 1. For molecular solids with a nonprimitive basis, the coupling of the different molecules causes a distinct excitonic spectral splitting of the absorption band (Davydov splitting) into an upper and a lower Davydov component, UDC and LDC, respectively. The pronounced coupling between equivalent molecules forming stacks causes an overall spectral shift of both Davydov components relative to the monomer absorbance in solution, as discussed previously in ref (33) and for completeness shown in Figure S1. With that, the monoclinic polymorph can be described as an H-type aggregate (overall spectral blue-shift) and the orthorhombic polymorph as a J-type aggregate (overall spectral red-shift).

The polarized absorbance properties in normal incidence transmission of crystalline textured thin film samples are then determined by the projection of the directions of the respective Davydov transitions onto the substrate plane.^33^ The yellow planes in Figure 1b and c visualize the molecular arrangement within the (110) and (011) planes, which have been observed previously for the two SQIB polymorphs spin-casted on nontemplating glass to be parallel to the surface. Here, the polymorph formation is controlled by thermal postannealing of the samples as discussed earlier.^33,42^

In other work, also the choice of solvent and amorphous interfacial coatings on the substrates have been found, in addition to annealing temperature, to be influential on the polymorph formation but without changing the out-of-plane orientation.^34^ Those results have been obtained for spin-casting of blended solutions containing a soluble fullerene acceptor such as PCBM, which does not alter the crystallization propensity and crystallographic orientation. This is consistent with our results for spin-casting PCBM-blended solutions on substrates with amorphous coatings such as MoO_3_^26,27^ and PEDOT:PSS^27^ typically used as interfacial layers in photovoltaic devices. Subsequent thermal annealing at 180 °C of a SQIB:PCBM blend with a 1:1 ratio by weight spin-casted on Indium Tin Oxide (ITO) results in concomitant polymorphs but favors the orthorhombic one.^30,31^ Detailed inspections by AFM and TEM cross-section imaging revealed the formation of a bilayer structure.^31^ A phase separation happens during annealing, and the PCBM sinks to the bottom and leaves characteristic elongated holes behind in the orthorhombic platelets forming on top. See also Table 1 for an overview of previously obtained results.

**Table 1:**
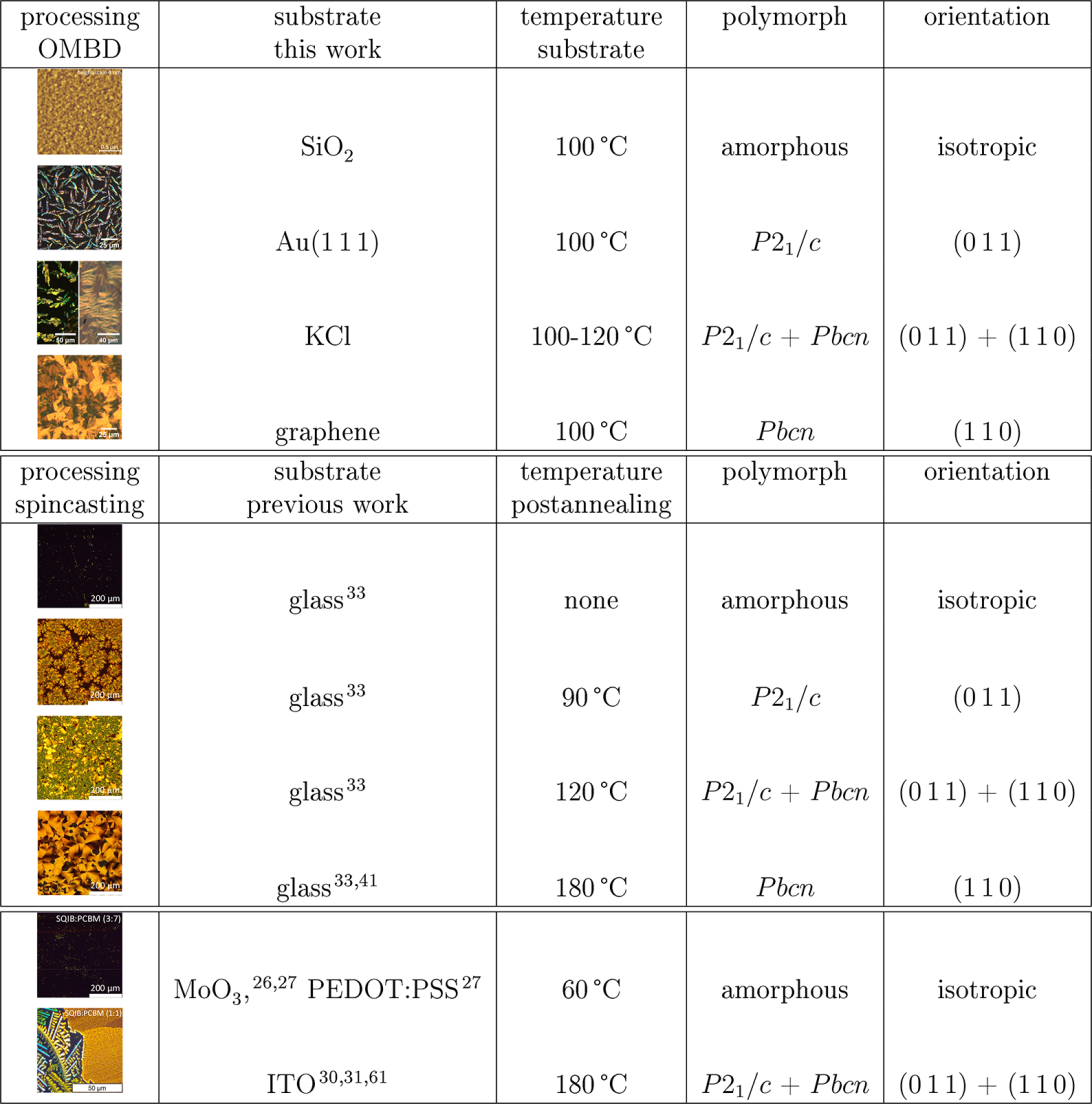
Summary of Polymorph Selection by Templating of Vapor-Deposited SQIB Thin Films (This Work) and by Postannealing of Solution-Processed Thin Films (Previous Works)^a^

a All images are optical microscopy images between crossed polarizers except for SQIB on SiO_2_, which is an AFM image. *P*2_1_/*c* = monoclinic polymorph, *Pbcn* = orthorhombic polymorph. Spin-casting SQIB:PCBM blends on MoO_3_, PEDOT:PSS, and ITO gives the same results as spin-casting on glass, which are not shown.

SQIB is one among the rare examples of donor-type semiconductors that can equifeasible be deposited from solution and by thermal vapor deposition. Also, codeposition with a fullerene acceptor such as C_60_ and C_70_ is possible; thereby, the study of photovoltaic device performance comparing solution and vacuum-processed bulk-heterojunctions was demonstrated.^28^ For both processing strategies, the amorphous SQIB phase was favored using low thermal postannealing temperatures and substrate temperatures during vapor deposition of the nontemplating MoO_3_ and PEDOT:PSS interfacial layers.

In this work, the focus is on polymorph selection, (out-of-plane) orientation and (in-plane) alignment control via templating of vapor-deposited SQIB. To achieve a more complete understanding of the diverse growth and structure formation of SQIB films, we have expanded previous work to allow a meaningful comparison. For this purpose, SQIB films have been deposited via organic molecular beam deposition (OMBD) on various dielectric and conductive substrates at elevated temperatures: silicon dioxide, potassium chloride, graphene, and gold showing weak, intermediate, and strong molecule–substrate interactions, respectively. Thereby, we show that vapor phase heterogeneous nucleation on different substrates (templating) allows polymorph selection including a postannealing-resistant amorphous isotropic thin film phase. By contrast, solution-processed SQIB films are initially amorphous and isotropic but allow a temperature-controlled polymorph selection by a postdeposition thermal annealing procedure. Crystallization happens on a seconds to minutes time-scale suitable for microscopic in situ monitoring. As intramolecular interactions exceed the molecule–substrate interactions for all growth substrates, the same film orientations occur for the two polymorphs formed in the annealed and vapor-deposited films, while the in-plane alignment is to some extent templated by the substrate.

## 2 Results and Discussion

SQIB thin films with nominal thicknesses of 30 nm have been obtained by OMBD on SiO_2_ (silicon wafer with native oxide), on a (111) gold layer supported by a mica substrate, on a graphene layer supported by a quartz substrate, and on a freshly cleaved (001) surface of a KCl single crystal. To enhance the crystalline order, the substrates have been heated to 100 °C during deposition, except for KCl, which was also heated to 120 °C. The deposition rate was 0.1 Å/s for all systems. SQIB is a thermally stable molecule because of the intermolecular hydrogen bonds from the hydroxy groups at the anilino ring and the squaric oxygen, so it can be vapor deposited without decomposition.^28,43^ This is verified by near edge X-ray absorption fine structure spectroscopy (NEXAFS), see Figure S2, showing the same characteristic NEXAFS signature for evaporated films and the raw powder. For comparison, additional SQIB films are prepared by spin-casting of chloroform solutions onto glass substrates, and the temperature-induced crystallization process is monitored in situ time-resolved by polarized optical microscopy.

**Figure 2:**
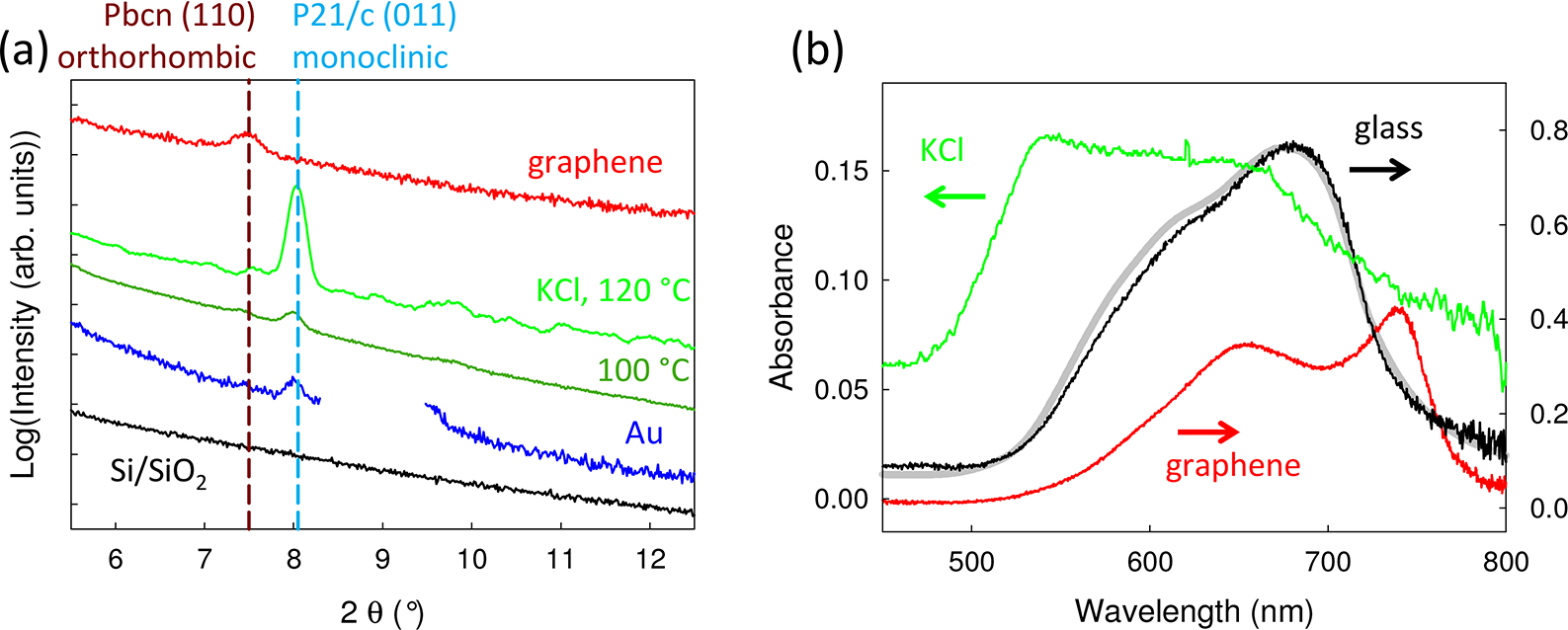
(a) Specular X-ray diffractograms are measured with Cu Kα radiation of SQIB on various substrates. Both polymorphs are found: the orthorhombic *Pbcn* and the monoclinic *P*2_1_/*c* phase depending on the growth substrate. The calculated positions for the (110) and (011) reflections are shown as dashed vertical lines. Note that for SQIB films grown on Au(111)/mica, a dominating mica peak at 2θ ≈ 9° was omitted. Vapor-deposited SQIB films on SiO_2_ as well as nonannealed, spin-casted films on glass (not shown) are XRD-silent. (b) Absorbance spectra measured in normal incidence transmission of nonannealed spin-casted (glass) and evaporated (graphene, KCl) SQIB thin films. The gray line is the absorbance calculated on the basis of the complex refractive index obtained from amorphous, isotropic SQIB films grown on SiO_2_ by ellipsometry as shown in Figure 5b.

Specular X-ray diffractograms as depicted in Figure 2a allow one to identify the adopted polymorph and the orientation of the films grown on various substrates. By contrast, vapor-deposited SQIB layers on SiO_2_ (black line) as well as nonannealed, spin-casted SQIB films on glass (not shown in the graph, see ref (33)) are XRD-silent, which indicates the presence of an amorphous isotropic phase. For the crystalline films, two diffraction peaks can be identified, one at 2θ = 7.6° corresponding to the (110) plane of the *Pbcn* phase, and another one at 2θ = 8.1° corresponding to the (011) plane of the *P*2_1_/*c* phase.^33^ On Au(111)/muscovite mica (blue line), (011) oriented films of the monoclinic phase are formed, while on graphene (red line), the orthorhombic phase with (110) orientation is clearly prevailing. On KCl(001), both polymorphs can be identified, but the monoclinic phase with (011) orientation is dominating (green lines). Raising the surface temperature during deposition from 100 °C (dark green line) to 120 °C (light green line) augments the monoclinic phase formation, and the overall XRD signal intensity increases. Therefore, 120 °C is chosen for discussion of the KCl(001) growth substrate.

For both realized crystallographic orientations, the molecular π-stacking direction ([001] for the orthorhombic polymorph, [100] for the monoclinic one) is parallel to the substrate; see also Figure 1. This is caused by the strong intermolecular interactions given by the slipped-π-stacking of the D–A–D backbones. These interactions in the crystalline bulk phase of thin films exceed the molecule–substrate interactions on the presently investigated inert substrates. Interestingly, we have found that the adopted polymorph can be selected by choice of the substrate, but find only distinct orientations on all investigated substrates ensuring the molecular face-to-face π-stacking direction to run parallel to the surface.

In Figure 2b, the unpolarized absorbance spectra (Abs = −log *T*, *T* normal incidence transmission) of the two SQIB polymorphs with their specific orientation as well as an amorphous, isotropic SQIB film are compared. The amorphous phase on glass (black line) has a broad absorbance peaking at 670 nm with a vibronic shoulder around 615 nm. The latter is in close agreement with the spectrum calculated (gray line) using the complex refractive index data obtained by ellipsometry from a film evaporated on SiO_2_. The (011)-orientated monoclinic phase found on KCl has a blue-shifted absorbance with a broad absorption from 530 to 630 nm (green line). By contrast, the absorbance is red-shifted for the (110)-oriented films of the orthorhombic polymorph found on graphene with a well-resolved Davydov splitting of 0.23 eV, peaking at 652 and 740 nm (red line). Because of such characteristic differences in the spectral signatures, simple UV/vis spectroscopy allows one to distinguish conclusively between the amorphous and the two crystalline SQIB phases. Polarized spectro-microscopy then allows mapping of samples containing concomitant polymorphs and also provides information about the local in-plane orientation. In the following, the impact of the substrate on the respective polymorph formation and the alignment is elucidated in more detail. Initially, a fresh in situ perspective on already known postannealing polymorph control of solution-processed films is presented.

### 2.1 Spin-Coated SQIB on Glass

Spin-casted thin films of SQIB on bare and indium tin oxide (ITO) coated glass have already been reported before.^31,33,41^ Chloroform was used as a rapidly evaporating solvent. Upon subsequent thermal annealing, depending on the annealing temperature, the two previously described polymorphs are found to various extents. Such samples have already been well characterized by XRD and polarized spectro-microscopy after the annealing step was finished. Here, for the first time, we monitor in situ the polymorph formation process during annealing at two characteristic temperatures. Time-resolved polarized optical microscopy movies from both crystallization processes are provided in the Supporting Information as movies S1 and S2.

**Figure 3:**
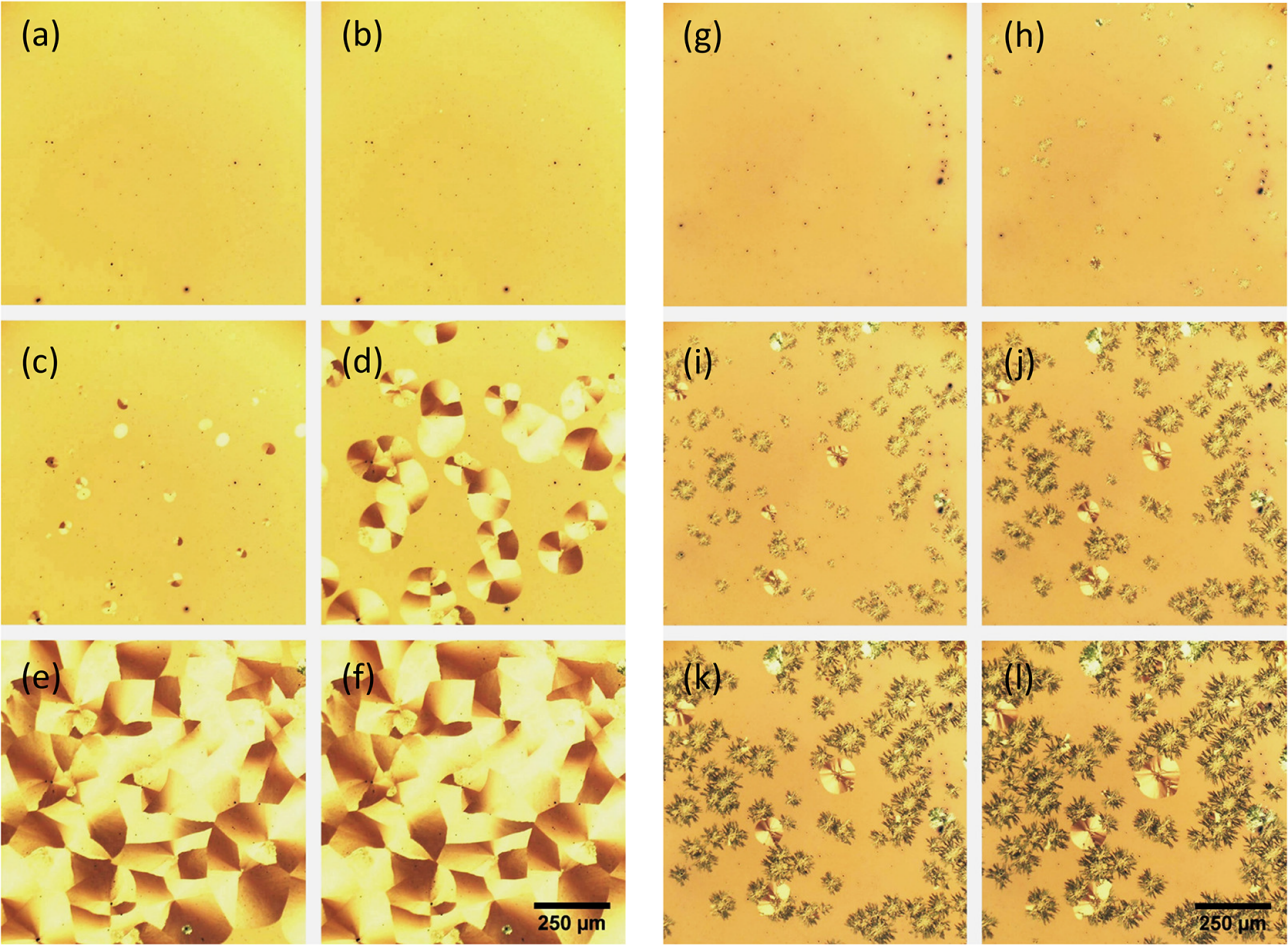
Polarized optical reflection microscope images (single polarizer) of spin-casted SQIB thin films onto a glass substrate show the formation of orthorhombic phase platelets (a–f) and monoclinic phase crystallites (g–l) during heating. For the platelets, images have been extracted from the movie at times (a) 2.4 s, (b) 2.9 s, (c) 3.5 s, (d) 4.1 s, (e) 4.6 s, and (f) 5.2 s after the sample was placed on a hot plate with a surface temperature of 195 °C. For the monoclinic phase crystallite formation, similar snapshots are taken at the times (g) 4 min, (h) 15 min, (i) 26 min, (j) 37 min, (k) 49 min, and (l) 60 min after the sample was placed on a hot plate with a surface temperature of 80 °C.

In Figure 3a–f, a time series of polarized optical micrographs extracted from the movies are shown. Here, the amorphous SQIB film spin-casted on glass is placed on a hot plate preheated to a surface temperature of 195 °C. Within seconds, platelets of the orthorhombic polymorph appear. When those start touching each other, domain boundaries are formed. AFM scans across a domain boundary between adjacent domains reveal a characteristic gap and allow one to determine the effective film/platelet thickness of around 50 nm for this sample; see Figure S3. After less than 10 s, the amorphous film transforms completely into a (110)-oriented polycrystalline film.

The extended orthorhombic phase domains are not single crystalline, but yet they are well suited for polarized spectro-microscopy investigation as previously published.^31,33^ For completeness, such an analysis is shown in Figures S4 and S5 for transmission and reflection. The complete diagonal dielectric tensor of SQIB could be determined by imaging ellipsometry, well reproducing the Davydov splitting.^41^ For completeness, the complex refractive indices along the three crystallographic axes are shown in Figure S6.

After a similar sample is placed onto a hot plate with a lower temperature such as 80 °C, as shown in Figure 3g–l, fibrous crystallite domains of the monoclinic polymorph evolve as growing from randomly distributed seeds. Note that also here a few platelets of the orthorhombic phase form. However, the time scale is much larger than for the formation of the orthorhombic polymorph in Figure 3a–f. The whole process lasts several hours until the entire, initially amorphous film is crystallized.

For intermediate annealing temperatures, both polymorphs crystallize to various extents on the surface. That way, spin-casted thin films can be crystallized into the desired polymorph or into specific ratios of concomitant polymorphs steered by the postannealing temperature.

**Figure 4:**
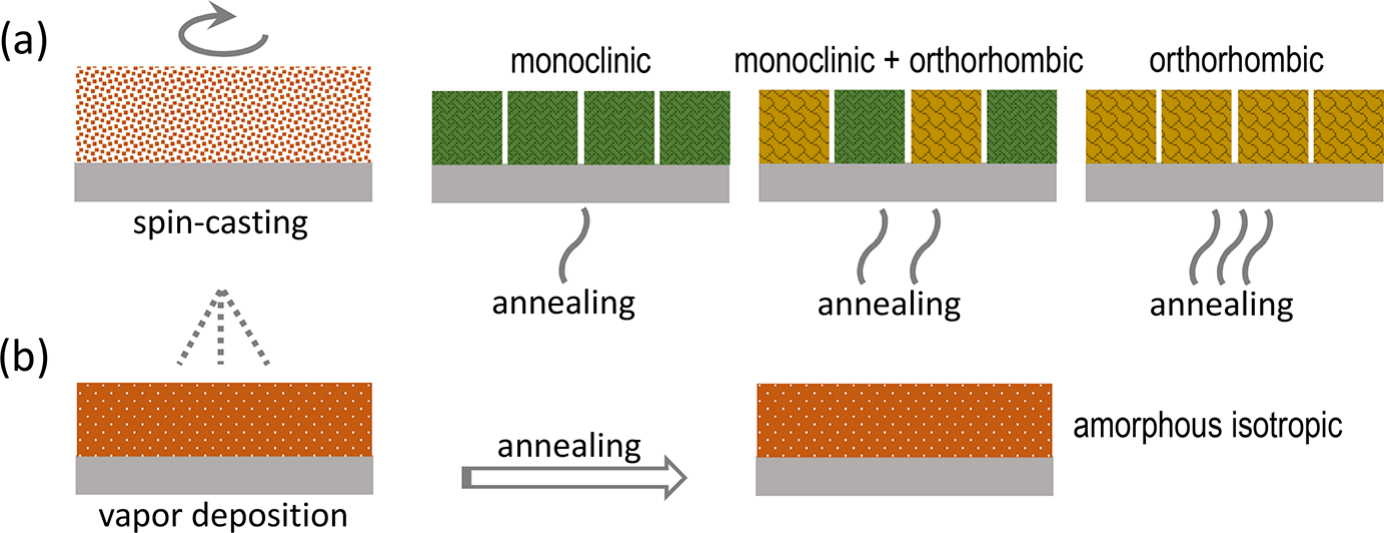
(a) Spin-casting of SQIB from chloroform likely gives less densely packed amorphous thin films containing voids due to fast solvent evaporation. Subsequent thermal annealing at around 90, 120, and 180 °C results in an excessive monoclinic phase, concomitant polymorphs, and excessive orthorhombic phase formation, respectively. (b) Vapor deposition presumably leads to more densely packed thin films without room for recrystallization upon postannealing.

This selective recrystallization is at least true for using chloroform as solvent for casting of the SQIB films. This rapidly evaporating solvent gives little time for the molecules to arrange into an equilibrium state.^44−46^ In these amorphous films, voids appear due to evaporated solvents, which allow for reorganization and thermally activated recrystallization.^45^ It is important to place the spin-casted films on a preheated hot plate at the specified temperatures to obtain the desired polymorph; see Figure 4a. By contrast, heating a sample from room temperature leads to excessive formation of the monoclinic polymorph (data not shown). Evolution of the orthorhombic polymorph appears to rely on kinetic control, that is, instant high temperature and rapid crystallite formation. Furthermore, transformation of the monoclinic to the orthorhombic phase by an additional annealing step could not be seen. These observations give the idea that the monoclinic polymorph is the thermodynamically stable phase, which is supported by the density rule. The density rule predicts that for van-der-Waals dominated polymorphs, the higher density polymorph is thermodynamically more stable.^10,17,35,36^ The calculated densities are ρ_*P*2_1_/*c*_ = 1.245 g/cm^3^ and ρ_*Pbcn*_ = 1.240 g/cm^3^, which give an approximately 0.5% higher density for the monoclinic polymorph. This suggests that for SQIB the monoclinic phase is the thermodynamically more stable one.

Furthermore, the size of the crystalline domains is regulated by the (random) occurrence of crystallization seeds, such as colloidal aggregates within the solution or dust particles.^47^ Also, spin-coating parameters affect the domain size in case of the formation of orthorhombic polymorph platelets: the higher is the acceleration, the larger the domains tend to be.^41^ However, the preferred crystalline orientation always remains the same for each polymorph driven by intermolecular interactions.

### 2.2 OMBD of SQIB on SiO_2_

**Figure 5:**
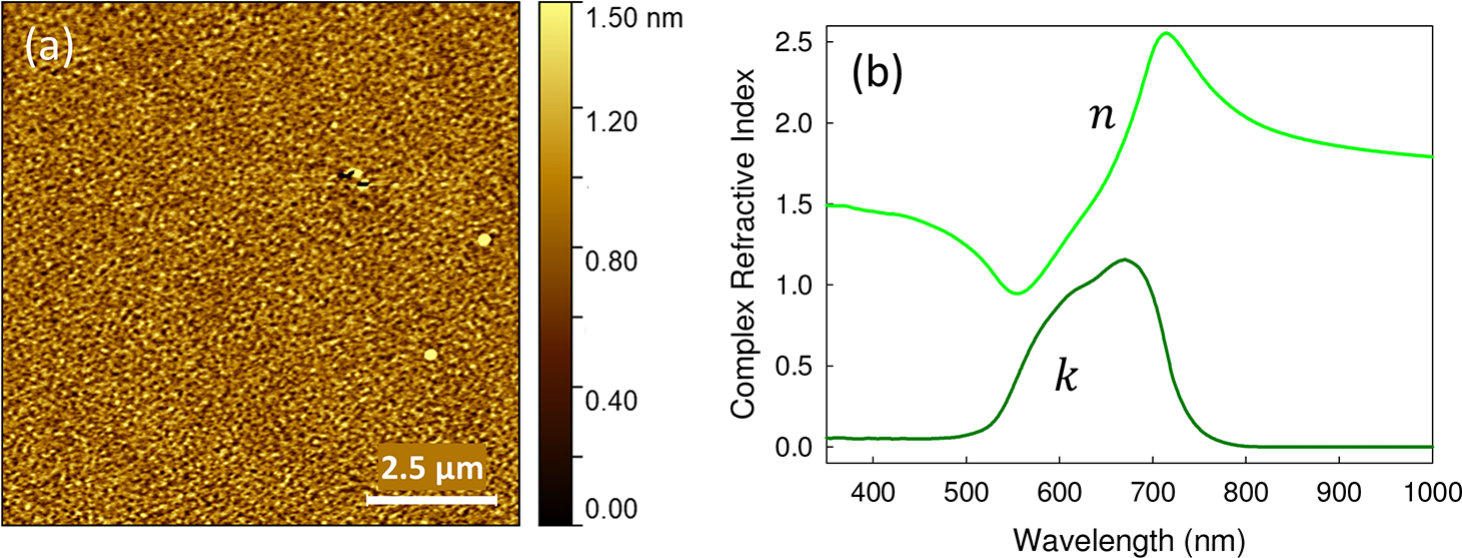
(a) AFM image and (b) real and imaginary parts of the complex index of refraction, *N* = *n* + *ik*, determined by variable angle spectroscopic ellipsometry of a SQIB film vapor deposited on SiO_2_ (Si-wafer covered with native oxide) at 100 °C substrate temperature.

For the vacuum deposition of SQIB on a Si-wafer with 2 nm native oxide at 100 °C, the X-ray diffractogram is featureless, which indicates that an amorphous thin film is formed, Figure 2b. Atomic force microscopy, Figure 5a, reveals that the film with a nominal thickness of 30 nm as derived from the quartz microbalance is very smooth with a rms roughness of 0.3 nm. Therefore, X-ray reflectivity (XRR) scans show well-resolved Kiessig fringes at small scattering angles, see Figure S1, which confirms the low surface roughness inferred by AFM. From such a smooth and extended thin SQIB film, the complex refractive index *N* = *n* + *ik* could easily be determined by standard spectroscopic ellipsometry confirming its isotropic nature, Figure 5b. Two samples with different layer thicknesses have been fitted simultaneously for improved reliability of the fit.^48^ For the sample shown in Figure 5a, ellipsometry determines the thickness to be 25 nm. A comparison between measured and calculated reflection spectra using OpenFilters^49^ for various angles of incidence shows a good agreement; see Figure S8. Likewise, the absorbance of nonannealed spin-coated SQIB films on glass is reproduced satisfactorily, Figure 2b. Interestingly, a thin SQIB film vapor deposited onto a silicon dioxide substrate has been reported by others to have a slight out-of-plane anisotropy.^50^ This hints to a dependence on processing parameters such as substrate temperature and deposition rate on the film formation. However, in both cases, the maximum of the extinction coefficient *k*, which quantifies the absorption capability, is easily exceeding 1, that is, exceeding 200 000 cm^–1^ at around 670 nm. This is a large value for amorphous organic semiconductor thin films. With that, such amorphous SQIB thin films are among the top candidates for light-harvesting organic photovoltaic materials.^51^ Even though all extinction coefficient values of the anisotropic tensor of the crystalline orthorhombic phase SQIB are larger by a factor of roughly 3,^41^ see above and Figure S6, the amorphous, isotropic phase is favored for macroscopic light-harvesting device application. This is due to the absence of polarization-dependent absorbance as well as domain boundaries as barriers for charge carriers.^52^ Nevertheless, local anisotropy can be beneficial for microscopic applications such as photovoltaic stimulation of living cells.^30,31^

Most remarkably, the vapor-deposited SQIB films on silicon are very stable in their amorphous isotropic phase in the present study. Neither growth at elevated substrate temperatures (here 100 °C) nor postdeposition annealing of the sample induces a notable crystallization. This is distinctly different for spin-coated films prepared at ambient temperatures on glass as discussed above. There, rapid evaporation of the chloroform solvent used for spin-casting is suspected to create less densely packed films leaving voids for subsequent thermally activated molecular reorganization.^44−46^ Here, in case of vapor deposition, the molecules are mobile on the substrate at elevated temperature and have more time to arrange into more densely packed solid films leaving no room for reorganization; see Figure 4b. Typically, vapor-deposited small molecular thin films are found to have a higher density and consequently are more heat-resisting (remaining in the initially formed phase) than are their solution-processed counterparts.^53^

### 2.3 OMBD of SQIB on Au(111)/Mica

**Figure 6:**
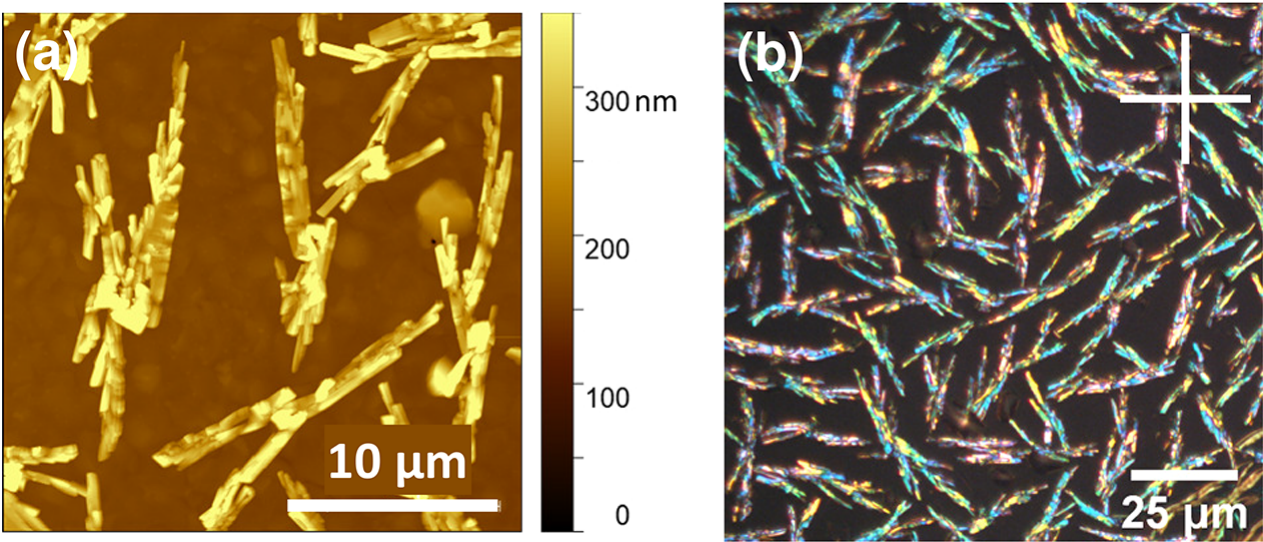
(a) AFM image of SQIB on Au(111), nominal thickness of 30 nm. A corresponding optical microscope image (reflection, crossed polarizers indicated by horizontal and vertical white line) (b) provides a large-scale impression.

Next, the vacuum deposition of a nominally 30 nm thick SQIB layer on the metallic substrate Au(111)/mica is investigated. Here, a film of the monoclinic phase with the (011) orientation is formed as deduced from XRD, Figure 2a. This means that molecular face-to-face π-stacking parallel to the surface is also favored on a metallic surface, which is typically strongly interacting with organic molecules. However, it has been found for the prototypical compound pentacene that it is actually a challenge to grow edge-on stacks on metallic surfaces, because after a few monolayers the molecules tend to relax into phases with upright standing molecules.^54,55^ Indeed, a submonolayer coverage of SQIB on silver surfaces showed the formation of a face-on wetting layer.^56^ In Figure 6a and b, an AFM image as well as a reflection microscopy image using crossed polarizers are shown. The large height of the fibrous elongated crystallites, about 10 times higher than the nominal film thickness, hints to dewetting. The colorful impression of the fibrous crystallites demonstrates the polycrystalline nature with small domain sizes similar to the annealing-induced monoclinic phase on glass substrates. While on glass the fibrous crystallites tend to grow away from a crystallization seed resulting in a dense, flower-like morphology, here separated elongated fibrous aggregates are formed. Similar to the glass sample, also for the monoclinic phase on gold the crystalline domain size is too small to be analyzed by the polarized spectro-microscopy setup. Within this survey, only on KCl(001) does the monoclinic SQIB phase grow into large enough domains suitable for further optical analysis as discussed in the following.

### 2.4 OMBD of SQIB on KCl(001)

**Figure 7:**
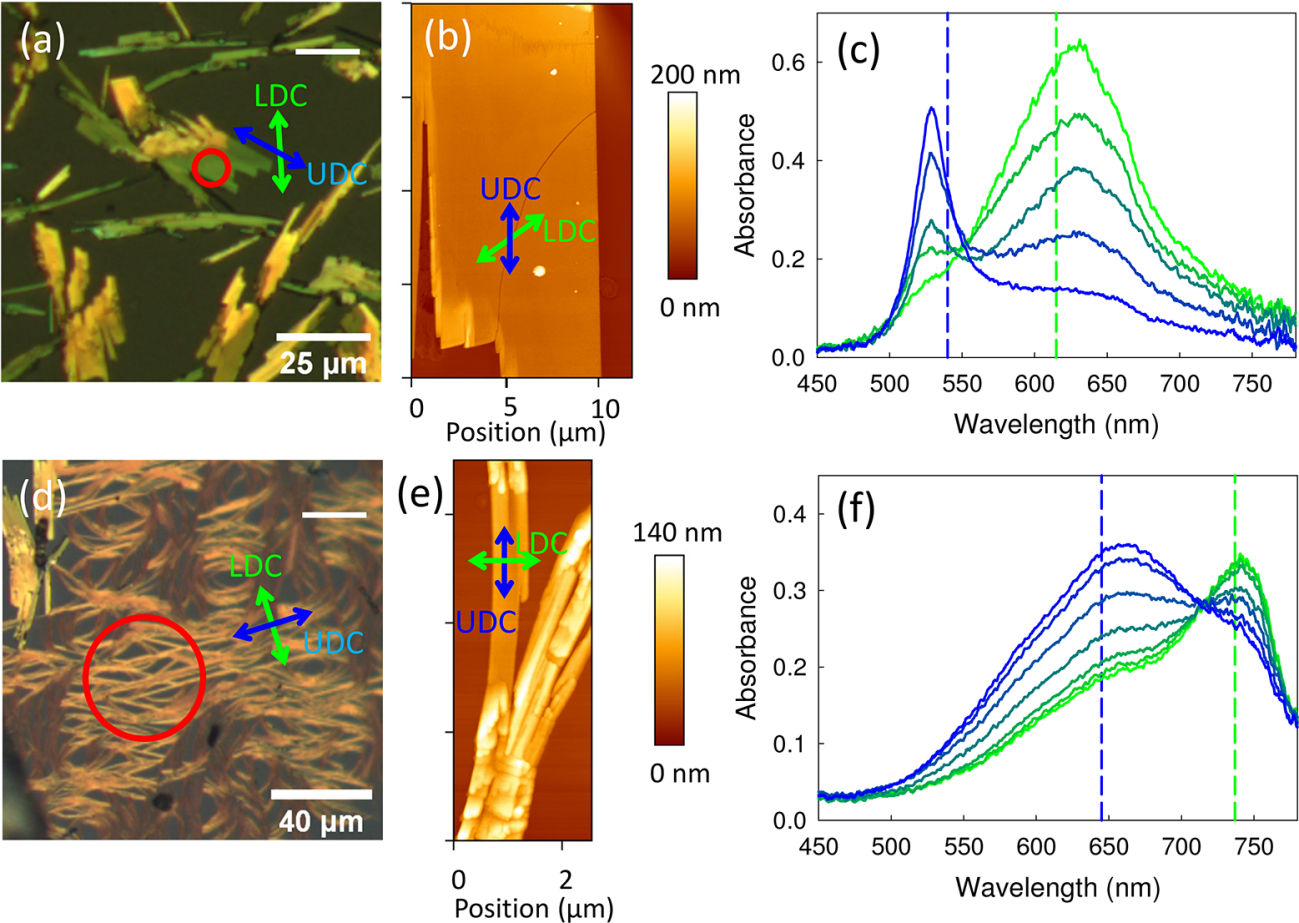
Polarized reflection microscopy images (single horizontal polarizer indicated by a white horizontal line) of SQIB evaporated onto KCl(001) at a substrate temperature of 120 °C, forming crystallites of the monoclinic phase (a) and fibers of the orthorhombic phase (d). AFM images of crystallites and fibers, (b) and (e), respectively. Corresponding polarized absorbance spectra, (c) and (f), demonstrate that the aggregates are constituted of different polymorphs. The dashed vertical lines mark the spectral positions of the maxima for spin-casted SQIB films on glass. For the spectra, the sample has been rotated over 60 and 90°, respectively, in steps of 15°. The red circles in the microscope images mark the position, where the absorbance spectra have been taken. The UDC and LDC directions are depicted in the microscope images by blue and green arrows, respectively.

For the thermal deposition of SQIB on freshly cleaved and annealed KCl(001) at an elevated substrate temperature of 120 °C, X-ray diffraction in Figure 2b hints to an excessive formation of the (011)-oriented monoclinic polymorph with minor regions of the (110)-oriented orthorhombic phase. The increased temperature (instead of 100 °C) has been chosen in case of KCl because the selective polymorph formation is more pronounced, and the crystallite size is larger allowing better inspection by polarized spectro-microscopy; see Figure S9. The morphology, however, is quite different from that of the previously described systems. Optical microscopy and AFM, Figure 7a,b and also Figures S10 and S11, show the predominant existence of elongated crystallites with a flat surface, but also spaghetti-like fibers appear, Figure 7d,e. An assignment of the polymorphic phase can safely be done by polarized spectro-microscopy due to its spatially resolving nature.

The flat crystallites appear green to yellow imaged between crossed polarizers with the color impression depending on the orientation of the crystallites. Polarized absorbance spectra as shown in Figure 7c confirm that the crystallites consist of the monoclinic polymorph. Here, two individual peaks are clearly resolved, while unpolarized absorbance measurements show a broad absorbance band with a flat plateau, Figure 2b. The Davydov splitting energy of the monoclinic phase on KCl amounts to 0.37 eV, which is larger as compared to the monoclinic phase formed by postannealing on glass substrates being 0.28 eV: UDC at 529 nm (540 nm on glass) and LDC at 629 nm (615 nm on glass). The angle Δ between polarizer orientations for the maxima of the UDC and LDC for the monoclinic polymorph is around 60 ± 6°, Figure S11c, whereas for the orthorhombic polymorph the angle is Δ = 90°, Figure S4. This value comes close to the expected angle of 56° for the case of the monoclinic phase.^33^

To a minor extent also spaghetti-like fibers are present, Figure 7d. These fibers can clearly be identified to consist of the orthorhombic polymorph by their absorbance spectrum, Figure 7f. As compared to the platelet spectra on glass, Figure S4d, the peaks are broader but have a slightly smaller Davydov splitting energy of 0.2 eV: UDC at 662 nm (645 nm on glass) and LDC at 743 nm (743 nm on glass). The peak broadening might be related to the fact that not a single fiber is measured but the absorbance is averaged over several fibers not fully parallel aligned within the field of view. The polarization angle difference Δ between maxima of the UDC and LDC for the fibers amounts to 90 ± 3°, that is in full agreement with the polarization behavior predicted by the molecular exciton theory,^39^ which has already been confirmed for the (110)-oriented orthorhombic polymorph platelets on glass, Figure S5.^33,41^ Here, the extended fiber-like shape on KCl(001) instead of the platelet shape suggests an epitaxial alignment of the orthorhombic phase with one of the KCl high symmetry directions during the growth process. That means, while the orientation still is dictated by the intermolecular interactions, the alignment is to some extent controlled by a templating effect of the KCl(001) substrate.

Because of the microsized crystalline texture of the discontinuous thin film, the direction of maximum reflectivity or minimum absorbance can be correlated with the crystallite/fiber orientations.^57^ Polarization analysis plots and histograms are shown in Figure S12 for the maximum reflectivity at λ = 650 ± 5 nm, selected by an interference filter inserted into the microscope. This probes both the LDC of the monoclinic phase crystallites and the UDC of the orthorhombic phase spaghetti-like fibers. The direction of UDC for the monoclinic phase crystallites and that of LDC for the orthorhombic phase fibers have been extracted from the local polarized transmission spectra, Figure S11. For the monoclinic phase crystallite, the UDC is found to be polarized along the long crystallite axis as indicated by the blue arrows in Figure 7a,b. From the correlation of the LDC direction with the long crystal axis, Figure S12b, a specific mean value of polarization direction for maximum reflectivity relative to the long crystallite direction of |β| = 58 ± 4° is obtained. The direction of the LDC is depicted by green arrows in Figure 7a,b. This is consistent with analysis of the spatially resolved polarized absorbance spectra, Figure S11. For the orthorhombic phase spaghetti-like fibers, the UDC is likewise polarized along the long fiber axis as indicated by blue arrows in Figure 7d,e. The green arrows depict the direction of the LDC, being rotated by 90° and therefore oriented along the short fiber axis.

### 2.5 OMBD of SQIB on Graphene/Quartz

**Figure 8:**
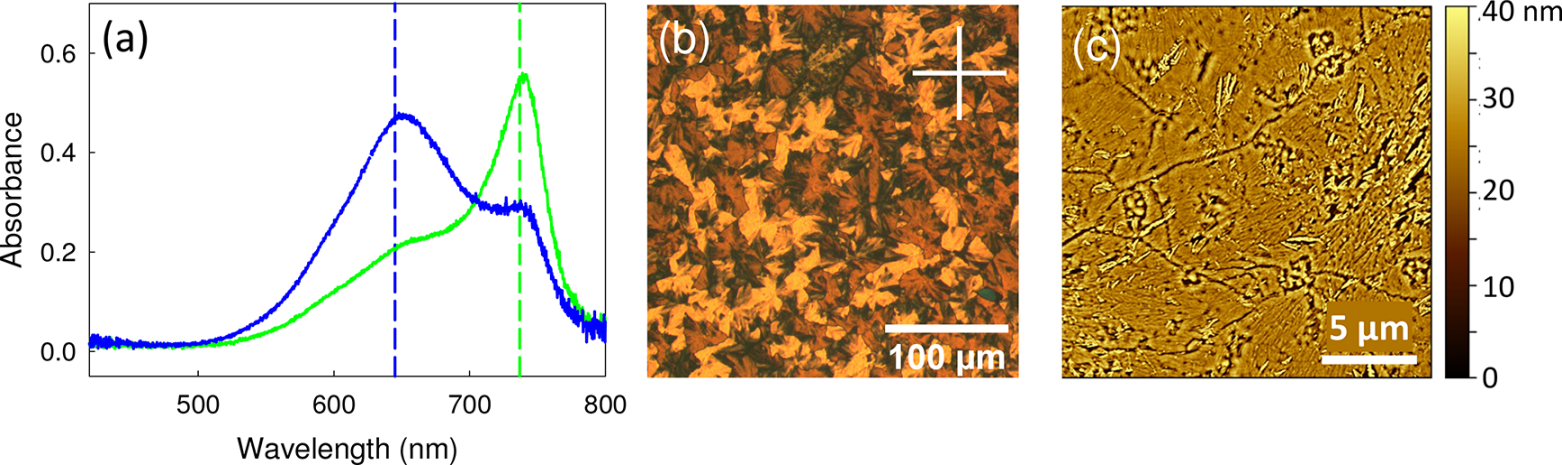
(a) Polarized absorbance spectra of a single SQIB domain, grown on graphene on quartz. The polarizer angle has been rotated by 90° between the two measurements. The positions of the two maxima agree well with the case of SQIB platelets on glass, dashed vertical lines. Both (b) polarized optical reflection microscopy (crossed polarizers, polarizer positions indicated by white cross) as well as the AFM micrograph (c) of platelets on graphene reveal an appearance similar to that for orthorhombic phase platelets on glass; see Figure S4.

For a nominally 30 nm thick OMBD grown film on graphene/quartz, deposited at a substrate temperature of 100 °C, (110)-oriented films of the orthorhombic phase form exclusively. While strong molecule–substrate interactions on metallic surfaces have been inferior to induce edge-on orientation in the case of pentacene,^54,55^ graphene and graphite offering intermediate molecule–substrate interactions emerged as templating substrates of choice to obtain edge-on oriented phases of phthalocyanines and pentacene.^58,59^ However, in case of SQIB, again the intermolecular interactions are decisive, and the typical (110)-orientation of the orthorhombic phase is expressed. The polarized absorbance spectra in Figure 8a show that the two absorbance bands visible in the unpolarized spectra, see Figure 2b, red curve, are polarized mutually perpendicular within the plane of the thin film. The complete polarization analysis is shown in Figure S13 and confirms that the optical absorbance properties are very similar to the orthorhombic phase platelets on glass, Figure S4. Just the peak maxima vary slightly: the UDC peaks at 652 nm (645 nm glass sample) and the LDC at 740 nm (737 nm glass sample) giving a Davydov splitting energy of 0.23 eV (0.24 eV glass sample). Also, the morphology determined by polarized optical microscopy as well as by AFM, see Figure 8b and c, is similar. Here, the domain size, however, is much smaller, dictated by the domain size of the graphene substrate. The uniformness of the optical image within these domains also suggests an epitaxial alignment, that is, a weak templating effect of the graphene substrate. This means that the platelets on graphene are rather single crystalline, mutually rotated domains.^60^ Platelets on nontemplating glass substrates can show a gradual rotation of the in-plane orientation within a single domain.^33,41^ This is evident from the gradual contrast change within a platelet image through a single polarizer, Figure 3d–f, or crossed polarizers, Figure S4b.

## 3 Conclusion

The anilino squaraine with iso-butyl terminal functionalization (SQIB) is a prototypical donor-type molecular semiconductor suitable for photovoltaic applications that can equifeasible be deposited by spin-casting and by thermal vapor deposition. The two known polymorphs of SQIB, a monoclinic and an orthorhombic phase, can be selected by heterogeneous nucleation from the gas phase (templating) on dielectric and conductive surfaces, such as silicon dioxide, potassium chloride, graphene, and gold, and by the postannealing temperature of solution-processed thin films; see the summary in Table 1. The rapid evaporation of the solvent is suspected to result in a metastable, less-densely packed molecular arrangement, leaving room for thermal activation of molecular reorganization. This is especially interesting for applications that desire a controlled crystalline microscopic patterning. For the vacuum-deposited thin films at a specific deposition temperature, the polymorph is templated by the growth substrate. For a nontemplating substrate, here a native-SiO_2_-coated wafer, a stable amorphous and isotropic phase is formed that cannot be recrystallized by subsequent thermal treatment. This is likely due to higher mobility of the adsorbed molecules on the growth substrate at elevated temperature allowing for a densely packed arrangement. Because the amorphous phase is favored for light-harvesting applications, which need to tolerate elevated device operation temperatures, vapor deposition appears to be the preferred processing technique. The three phases of SQIB (amorphous, monoclinic, and orthorhombic) can easily be distinguished by their absorbance spectra showing Frenkel excitonic signatures and their birefringent appearance. The pronounced molecular interactions, characteristic for squaraine compounds due to their D–A–D backbone, are dominating in the bulk phase of both polymorphs. Therefore, the same orientation is obtained for each polymorph on every substrate, so that the molecular stacking direction always runs parallel to the surface. While with that the out-of-plane orientation is fixed, the morphology and in-plane arrangement can to some extent be controlled by epitaxial alignment.

## 4 Materials and Methods

### 4.1 Sample Preparation

2,4-Bis[4-(*N*,*N*-diisobutylamino)-2,6-dihydroxyphenyl]squaraine (SQIB) has been synthesized following our previously published procedure.^61^ It crystallizes into two different polymorphs: single-crystal structures of the monoclinic *P*2_1_/*c* (CCDC code 1567209) and of the orthorhombic *Pbcn* phases (CCDC code 1567104) have been published earlier.^33^ For deposition via spin-coating, a 5 mg/mL solution of SQIB in chloroform (Sigma-Aldrich, stabilized with amylene) was prepared. The solution was spin-casted in inert atmosphere at 3000 rpm, ramping 3, for 60 s (SÜSS MicroTec LabSpin). This was followed by annealing on a preheated hot plate at the indicated surface temperatures for minutes to hours (IKA yellow line). The surface temperature was validated by a thermocouple contact thermometer.

In a vacuum (base pressure *p* = 1 × 10^–7^ mbar), samples are prepared by organic molecular beam deposition (OMBD) at a deposition rate of 0.1 Å/s. For this, the crucible is heated to 260 °C. A substrate temperature of 100 °C has been chosen for SiO_2_, graphene/quartz, and Au(111)/mica, but for KCl substrate the temperature was raised to 120 °C. Likewise, the same nominal thickness of 30 nm was also deposited for the sake of comparability. Note that the nominal thickness refers to the integrated signal of a quartz microbalance and the local true film thickness on the sample can deviate, especially for textured thin films.

### 4.2 In Situ Optical Characterization

Temperature-resolved optical microscopy was performed using an optical microscope in reflection geometry in combination with a heatable sample holder. The temperature was controlled using a K-type thermocouple that was placed directly next to the sample.

### 4.3 X-ray Diffraction

X-ray diffraction (XRD) on thin films was performed in Bragg–Brentano geometry with an automatic divergence slit. A PANalytical XPertPro MPD diffractometer using Cu Kα radiation (λ = 1.541 Å) was used, with the tube set to 40 kV and 40 mA with a 10 mm beam mask. Samples were rotated in a sample spinner during measurement to eliminate possible effects from preferential in-plane orientation. In addition, a Bruker D8 Discovery diffractometer using Cu Kα radiation was used.

### 4.4 Scanning Probe Microscopy

Morphological characterization took place by atomic force microscopy (AFM, JPK Nanowizard). Typically, intermittent contact images were taken with BudgetSensors Tap300-G tips with a force constant of 40 N/m, a resonance frequency 300 kHz, and a tip radius smaller than 10 nm. The AFM was situated on an inverted optical microscope (Nikon Eclipse TE 300) to allow simultaneous optical and morphological characterization. In addition, an Agilent SPM 5500 system was used operated in tapping mode with MikroMasch cantilevers, a resonance frequency of 325 kHz, and a spring constant of 40 N/m. Samples were imaged under ambient conditions. Gwyddion^62^ as well as the software provided by the AFM manufacturers have been used for image analysis.

### 4.5 Ellipsometry

The complex refractive index of isotropic films has been determined by variable angle spectroscopic ellipsometry utilizing a J.A. Woollam rotating analyzer ellipsometer (VASE) with vertical sample stage. Standard ellipsometric data in reflection as well as p-polarized reflection have been recorded with WVASE32 software. Parameters: AOI = 15°, 35°, 55°, AutoSlit = 1 mm, wavelength steps = 5 nm, wavelength range = 350–1700 nm. The ellipsometric data were converted to CompleteEASE (CE) format and analyzed with CE version 6 using Multi Sample Analysis to fit samples with various layer thicknesses simultaneously for decoupling of fit parameters.^48^ Here, two samples of 25 and 48 nm thickness have been analyzed. The layer thickness was determined within the transparent spectral range from 900 to 1700 nm by a Cauchy model. This was then converted to a model free, Kramers–Kronig-consistent isotropic B-spline fit and extended over the full spectral range with 0.1 eV resolution (except 660–720 nm with 0.05 eV resolution).

For the Si substrate with native oxide, the database complex refractive index “SI_JAW” fits well, and the native oxide layer was determined to be 2.02 nm thick using the “NTVE_JAW” database complex refractive index.

### 4.6 Polarized Optical Characterization

For the basic polarized optical characterization, a polarization microscope (Leitz DMRME) was used. The projected orientations of the upper and lower Davydov components within the thin films were determined by polarized reflection and transmission microscopy. Illumination took place either by linear polarized white light or by quasi-monochromatic light, selected by bandpass filters (Thorlabs FKB-VIS-10 and VEB Carl Zeiss Jena) with a fwhm of 10 and 7.5 nm, respectively. To determine, spatially resolved, the LDC and UDC directions, the sample was rotated in steps of 5° over 360° by a computer-controlled rotation stage. For each angle, a microscope image was taken. The series of images was analyzed in ImageJ^63^ by a discrete Fourier transform.^57,64^ From the intensity variation *I*^*x*,*y*^ of the pixel at position (*x*,*y*), the angle ϕ_1pol_(*x*,*y*) for the largest reflectivity or transmission is calculated. To correlate the polarization angle ϕ_1pol_(*x*,*y*) with the crystallite or fiber directions, their local orientation θ_orient_(*x*, *y*) at position (*x*,*y*) is determined with the help of the structure tensor.^64,65^ From this, the angle of maximum reflectivity at a certain wavelength with respect to the long crystallite direction, β = ϕ_1pol_ – θ_orient_, is determined. Spatially resolved, polarized transmission and reflection spectra were measured in a similar way with a fiber-optics miniature spectrometer (Ocean Optics Maya2000), coupled via a 200 μm diameter fiber to the camera port of the microscope.

## Supporting Information Available

Unpolarized absorbance spectra for SQIB on various substrates; NEXAFS data; AFM and optical microscope images for SQIB deposited on various substrates; polarized absorbance and reflectivity; components of the SQIB dielectric tensor; XRD data; measured and modeled reflection spectra for non-normal incidence; and polarization analysis (PDF)
Movie S1: Crystallization process of SQIB on glass (AVI)
Movie S2: Crystallization process of SQIB on glass (AVI)

## Supplementary Material

la2c01023_si_001.pdf

la2c01023_si_002.avi

la2c01023_si_003.avi

## References

[ref1] Kahr B.; Freudenthal J.; Gunn E. Crystals in Light. Acc. Chem. Res. 2010, 43, 684–692. 10.1021/ar900288m. 20180582

[ref2] Brewer J.; Schiek M.; Wallmann I.; Rubahn H.-G. First order optical nonlinearities χ^2^ for organic nanofibers from functionalized para-phenylenes. Opt. Commun. 2008, 281, 3892–3896. 10.1016/j.optcom.2008.03.075.

[ref3] Zablocki J.; Arteaga O.; Balzer F.; Hertel D.; Holstein J.; Clever G.; Anhäuser J.; Puttreddy R.; Rissanen K.; Meerholz K.; Lützen A.; Schiek M. Polymorphic Chiral Squaraine Crystallites in Textured Thin Films. Chirality 2020, 32, 619–631. 10.1002/chir.23213. 32155676

[ref4] Schulz M.; Zablocki J.; Abdullaeva O. S.; Brück S.; Balzer F.; Lützen A.; Arteaga O.; Schiek M. Giant Intrinsic Circular Dichroism of Prolinol-Derived Squaraine Thin Films. Nat. Commun. 2018, 9, 2413 10.1038/s41467-018-04811-7. 29925832 PMC6010436

[ref5] Llinás A.; Goodman J. M. Polymorph control: past, present and future. Drug Discovery Today 2008, 13, 198–210. 10.1016/j.drudis.2007.11.006. 18342795

[ref6] Hiszpanski A. M.; Baur R. M.; Kim B.; Tremblay N. J.; Nuckolls C.; Woll A. R.; Loo Y.-L Tuning Polymorphism and Orientation in Organic Semiconductor Thin Films via Post-deposition Processing. J. Am. Chem. Soc. 2014, 136, 15749–15756. 10.1021/ja5091035. 25317987

[ref7] Gentili D.; Gazzano M.; Melucci M.; Jones D.; Cavallini M. Polymorphism as an additional functionality of materials for technological applications at surfaces and interfaces. Chem. Soc. Rev. 2019, 48, 2502–2517. 10.1039/C8CS00283E. 30869083

[ref8] Jones A. O. F.; Chattopadhyay B.; Geerts Y. H.; Resel R. Substrate-Induced and Thin-Film Phases: Polymorphism of Organic Materials on Surfaces. Adv. Funct. Mater. 2016, 26, 2233–2255. 10.1002/adfm.201503169.

[ref9] Cruz-Cabeza A. J.; Reutzel-Edens S. M.; Bernstein J. Facts and fictions about polymorphism. Chem. Soc. Rev. 2015, 44, 8619–8635. 10.1039/C5CS00227C. 26400501

[ref10] Bernstein J. Polymorphism in Molecular Crystals; Oxford University Press: New York, 2020.

[ref11] Shen C.-A.; Bialas D.; Hecht M.; Stepanenko V.; Sugiyasu K.; Würthner F. Polymorphism in Squaraine Dye Aggregates by Self-Assembly Pathway Differentiation: Panchromatic Tubular Dye Nanorods versus J-Aggregate Nanosheets. Angew. Chem. - Int. Ed. 2021, 60, 11949–11958. 10.1002/anie.202102183. PMC8252746 33751763

[ref12] Lee W.; Park J.; Sim S.; Lim S.; Kim K.; Hong B.; Cho K. Surface-Directed Molecular Assembly of Pentacene on Monolayer Graphene for High-Performance Organic Transistors. J. Am. Chem. Soc. 2011, 133, 4447–4454. 10.1021/ja1097463. 21381751

[ref13] Riera-Galindo S.; Tamayo A.; Mas-Torrent M. Role of Polymorphism and Thin-Film Morphology in Organic Semiconductors Processed by Solution Shearing. ACS Omega 2018, 3, 2329–2339. 10.1021/acsomega.8b00043. 29503976 PMC5830697

[ref14] Fan Z.-P.; Zhang H.-L. Integrated Circuits/Microchips; IntechOpen, 2020.

[ref15] Bischof D.; Zeplichal M.; Anhäuser S.; Kumar A.; Kind M.; Kramer F.; Bolte M.; Ivlev S. I.; Terfort A.; Witte G. Perfluorinated Acenes: Crystalline Phases, Polymorph-Selective Growth, and Optoelectronic Properties. J. Phys. Chem. C 2021, 125, 19000–19012. 10.1021/acs.jpcc.1c05985.

[ref16] Blagden N.; de Matas M.; Gavan P.; York P. Crystal engineering of active pharmaceutical ingredients to improve solubility and dissolution rates. Adv. Drug Delivery Rev. 2007, 59, 617–630. 10.1016/j.addr.2007.05.011. 17597252

[ref17] Lee E. H. A practical guide to pharmaceutical polymorph screening & selection. Asian J. Pharm. Sci. 2014, 9, 163–175. 10.1016/j.ajps.2014.05.002.

[ref18] Yang J.; Hu C. T.; Zhu X.; Zhu Q.; Ward M. D.; Kahr B. DDT Polymorphism and the Lethality of Crystal Forms. Angew. Chem. 2017, 129, 10299–10303. 10.1002/ange.201703028. 28608599

[ref19] Parambil J. V.; Poornachary S. K.; Heng J. Y.; Tan R. B. Template-induced nucleation for controlling crystal polymorphism: from molecular mechanisms to applications in pharmaceutical processing. CrystEngComm 2019, 21, 4122–4135. 10.1039/C9CE00404A.

[ref20] Prokhorov V. V.; Pozin S. I.; Perelygina O. M.; Mal’tsev E. I. Crystallography and Molecular Arrangement of Polymorphic Monolayer J-Aggregates of a Cyanine Dye: Multiangle Polarized Light Fluorescence Optical Microscopy Study. Langmuir 2018, 34, 4803–4810. 10.1021/acs.langmuir.8b01008. 29601203

[ref21] Halton B. From Small Rings to Big Things: Xerography Sensors, and the Squaraines. Chem. New Zealand 2008, 72, 57–62.

[ref22] Weiss D. S. The History and Development of Organic Photoconductors for Electrophotography. J. Imaging Sci. Technol. 2016, 60, 305051–3050524. 10.2352/J.ImagingSci.Technol.2016.60.3.030505.

[ref23] Chen G.; Sasabe H.; Igarashi T.; Hong Z.; Kido J. Squaraine dyes for organic photovoltaic cells. J. Mater. Chem. A 2015, 3, 14517–14534. 10.1039/C5TA01879J.

[ref24] Chen Y.; Zhu W.; Wu J.; Huang Y.; Facchetti A.; Marks T. J. Recent Advances in Squaraine Dyes for Bulk-Heterojunction Organic Solar Cells. Org. Photonics Photovolt. 2019, 6, 1–16. 10.1515/oph-2019-0001.

[ref25] Brück S.; Krause C.; Turrisi R.; Beverina L.; Wilken S.; Saak W.; Lützen A.; Borchert H.; Schiek M.; Parisi J. Structure–property relationship of anilino-squaraines in organic solar cells. Phys. Chem. Chem. Phys. 2014, 16, 1067–1077. 10.1039/C3CP54163K. 24288034

[ref26] Scheunemann D.; Kolloge O.; Wilken S.; Mack M.; Parisi J.; Schulz M.; Lützen A.; Schiek M. Revealing the recombination dynamics in squaraine-based bulk heterojunction solar cells. Appl. Phys. Lett. 2017, 111, 183502 10.1063/1.4996080.

[ref27] Scheunemann D.; Wilken S.; Sandberg O. J.; Österbacka R.; Schiek M. Effect of Imbalanced Charge Transport on the Interplay of Surface and Bulk Recombination in Organic Solar Cells. Phys. Rev. Applied 2019, 11, 054090 10.1103/PhysRevApplied.11.054090.

[ref28] Chen G.; Ling Z.; Wei B.; Zhang J.; Hong Z.; Sasabe H.; Kido J. Comparison of the Solution and Vacuum-Processed Squaraine:Fullerene Small-Molecule Bulk Heterojunction Solar Cells. Front. Chem. 2018, 6, 1 10.3389/fchem.2018.00412. 30255017 PMC6141623

[ref29] Schulz M.; Balzer F.; Scheunemann D.; Arteaga O.; Lützen A.; Meskers S.; Schiek M. Chiral Excitonic Organic Photodiodes for Direct Detection of Circular Polarized Light. Adv. Funct. Mater. 2019, 29, 1900684 10.1002/adfm.201900684.

[ref30] Abdullaeva O. S.; Balzer F.; Schulz M.; Parisi J.; Lützen A.; Dedek K.; Schiek M. Organic Photovoltaic Sensors for Photocapacitive Stimulation of Voltage-Gated Ion Channels in Neuroblastoma Cells. Adv. Funct. Mater. 2019, 29, 1805177 10.1002/adfm.201805177.

[ref31] Balzer F.; Abdullaeva O. S.; Maderitsch A.; Schulz M.; Lützen A.; Schiek M. Nanoscale Polarization-Resolved Surface Photovoltage of a Pleochroic Squaraine Thin Film. Phys. Status Solidi B 2020, 257, 1900570 10.1002/pssb.201900570.

[ref32] Momma K.; Izumi F. VESTA 3 for three-dimensional visualization of crystal, volumetric and morphology data. J. Appl. Crystallogr. 2011, 44, 1272–1276. 10.1107/S0021889811038970.

[ref33] Balzer F.; Kollmann H.; Schulz M.; Schnakenburg G.; Lützen A.; Schmidtmann M.; Lienau C.; Silies M.; Schiek M. Spotlight on Excitonic Coupling in Polymorphic and Textured Anilino Squaraine Thin Films. Cryst. Growth Des. 2017, 17, 6455–6466. 10.1021/acs.cgd.7b01131.

[ref34] Viterisi A.; Montcada N. F.; Kumar C. V.; Gispert-Guirado F.; Martin E.; Escudero E.; Palomares E. Unambiguous determination of molecular packing in crystalline donor domains of small molecule solution processed solar cell devices using routine X-ray diffraction techniques. J. Mater. Chem. A 2014, 2, 3536 10.1039/c3ta13116e.

[ref35] Burger A.; Ramberger R. On the polymorphism of pharmaceuticals and other molecular crystals. I. Mikrochim. Acta 1979, 72, 259–271. 10.1007/BF01197379.

[ref36] Burger A.; Ramberger R. On the polymorphism of pharmaceuticals and other molecular crystals. II. Mikrochim. Acta 1979, 72, 273–316. 10.1007/BF01197380.

[ref37] Davydov A. S. The theory of molecular excitons. Phys. Usp. 1964, 7, 145 10.1070/PU1964v007n02ABEH003659.

[ref38] Kasha M.; Rawls H.; El-Bayoumi M. The exciton model in molecular spectroscopy. Pure Appl. Chem. 1965, 11, 371–392. 10.1351/pac196511030371.

[ref39] Hestand N. J.; Spano F. C. Expanded Theory of H- and J-Molecular Aggregates: The Effect of Vibronic Coupling and Intermolecular Charge Transfer. Chem. Rev. 2018, 118, 7069–7163. 10.1021/acs.chemrev.7b00581. 29664617

[ref40] Breuer T.; Celik M. A.; Jakob P.; Tonner R.; Witte G. Vibrational Davydov Splittings and Collective Mode Polarizations in Oriented Organic Semiconductor Crystals. J. Phys. Chem. C 2012, 116, 14491–14503. 10.1021/jp304080g.

[ref41] Funke S.; Duwe M.; Balzer F.; Thiesen P. H.; Hingerl K.; Schiek M. Determining the Dielectric Tensor of Microtextured Organic Thin Films by Imaging Mueller Matrix Ellipsometry. J. Phys. Chem. Lett. 2021, 12, 3053–3058. 10.1021/acs.jpclett.1c00317. 33739845 PMC8041376

[ref42] Freese S.; Lässing P.; Jakob R.; Schulz M.; Lützen A.; Schiek M.; Nilius N. Photoluminescence of Squaraine Thin Films: Spatial Homogeneity and Temperature Dependence. Phys. Status Solidi B 2019, 256, 1800450 10.1002/pssb.201800450.

[ref43] Tian M.; Furuki M.; Iwasa I.; Sato Y.; Pu L.; Tatsuura S. Search for Squaraine Derivatives That Can Be Sublimed without Thermal Decomposition. J. Phys. Chem. B 2002, 106, 4370–4376. 10.1021/jp013698r.

[ref44] Ingrosso C.; Curri M. L.; Fini P.; Giancane G.; Agostiano A.; Valli L. Functionalized Copper(II)-Phthalocyanine in Solution and As Thin Film: Photochemical and Morphological Characterization toward Applications. Langmuir 2009, 25, 10305–10313. 10.1021/la9010955. 19485406

[ref45] Eccher J.; Zajaczkowski W.; Faria G. C.; Bock H.; von Seggern H.; Pisula W.; Bechtold I. H. Thermal Evaporation versus Spin-Coating: Electrical Performance in Columnar Liquid Crystal OLEDs. ACS Appl. Mater. Interfaces 2015, 7, 16374–16381. 10.1021/acsami.5b03496. 26168313

[ref46] Cranston R. R.; Lessard B. H. Metal phthalocyanines: thin-film formation, microstructure, and physical properties. RSC Adv. 2021, 11, 21716–21737. 10.1039/D1RA03853B. 35478816 PMC9034105

[ref47] Yu L.; Niazi M. R.; Ndjawa G. O. N.; Li R.; Kirmani A. R.; Munir R.; Balawi A. H.; Laquai F.; Amassian A. Programmable and coherent crystallization of semiconductors. Sci. Adv. 2017, 3, 1602462 10.1126/sciadv.1602462. PMC5336352 28275737

[ref48] Zablocki J.; Schulz M.; Schnakenburg G.; Beverina L.; Warzanowski P.; Revelli A.; Grüninger M.; Balzer F.; Meerholz K.; Lützen A.; Schiek M. Structure and Dielectric Properties of Anisotropic n-Alkyl Anilino Squaraine Thin Films. J. Phys. Chem. C 2020, 124, 22721–22732. 10.1021/acs.jpcc.0c07498.

[ref49] Larouche S.; Martinu L. OpenFilters: open-source software for the design, optimization, and synthesis of optical filters. Appl. Opt. 2008, 47, C219–C230. 10.1364/AO.47.00C219. 18449250

[ref50] Chen G.; Yokoyama D.; Sasabe H.; Hong Z.; Yang Y.; Kido J. Optical and electrical properties of a squaraine dye in photovoltaic cells. Appl. Phys. Lett. 2012, 101, 083904 10.1063/1.4747623.

[ref51] Vezie M. S.; Few S.; Meager I.; Pieridou G.; Dörling B.; Ashraf R. S.; Goñi A. R.; Bronstein H.; McCulloch I.; Hayes S. C.; Campoy-Quiles M.; Nelson J. Exploring the origin of high optical absorption in conjugated polymers. Nat. Mater. 2016, 15, 746–753. 10.1038/nmat4645. 27183327

[ref52] Zheng C.; Mark M. F.; Wiegand T.; Diaz S. A.; Cody J.; Spano F. C.; McCamant D. W.; Collison C. J. Measurement and Theoretical Interpretation of Exciton Diffusion as a Function of Intermolecular Separation for Squaraines Targeted for Bulk Heterojunction Solar Cells. J. Phys. Chem. C 2020, 124, 4032–4043. 10.1021/acs.jpcc.9b11816.

[ref53] Shibata M.; Sakai Y.; Yokoyama D. Advantages and disadvantages of vacuum-deposited and spin-coated amorphous organic semiconductor films for organic light-emitting diodes. J. Mater. Chem. C 2015, 3, 11178–11191. 10.1039/C5TC01911G.

[ref54] Käfer D.; Ruppel L.; Witte G. Growth of pentacene on clean and modified gold surfaces. Phys. Rev. B 2007, 75, 085309 10.1103/PhysRevB.75.085309.

[ref55] Zheng Y.; Qi D.; Chandrasekhar N.; Gao X.; Troadec C.; Wee A. T. S. Effect of Molecule-Substrate Interaction on Thin-Film Structures and Molecular Orientation of Pentacene on Silver and Gold. Langmuir 2007, 23, 8336–8342. 10.1021/la063165f. 17602678

[ref56] Luft M.; Groß B.; Schulz M.; Lützen A.; Schiek M.; Nilius N. Adsorption of squaraine molecules to Au(111) and Ag(001) surfaces. J. Chem. Phys. 2018, 148, 074702 10.1063/1.5017826. 29471637

[ref57] Balzer F.; Schiek M.; Osadnik A.; Wallmann I.; Parisi J.; Rubahn H.-G.; Lützen A. Substrate Steered Crystallization of Naphthyl End-Capped Oligothiophenes into Nanowires: The Influence of Methoxy-Functionalization. Phys. Chem. Chem. Phys. 2014, 16, 5747–5754. 10.1039/c3cp53881h. 24531698

[ref58] Xiao K.; Deng W.; Keum J. K.; Yoon M.; Vlassiouk I. V.; Clark K. W.; Li A.-P.; Kravchenko I. I.; Gu G.; Payzant E. A.; Sumpter B. G.; Smith S. C.; Browning J. F.; Geohegan D. B. Surface-Induced Orientation Control of CuPc Molecules for the Epitaxial Growth of Highly Ordered Organic Crystals on Graphene. J. Am. Chem. Soc. 2013, 135, 3680–3687. 10.1021/ja3125096. 23368998

[ref59] Götzen J.; Käfer D.; Wöll C.; Witte G. Growth and structure of pentacene films on graphite: Weak adhesion as a key for epitaxial film growth. Phys. Rev. B 2010, 81, 085440 10.1103/PhysRevB.81.085440.

[ref60] Balzer F.; Henrichsen H.; Klarskov M.; Booth T.; Sun R.; Parisi J.; Schiek M.; Bøggild P. Directed self-assembled crystalline oligomer domains on graphene and graphite. Nanotechnology 2014, 25, 035602 10.1088/0957-4484/25/3/035602. 24356510

[ref61] Abdullaeva O. S.; Schulz M.; Balzer F.; Parisi J.; Lützen A.; Dedek K.; Schiek M. Photoelectrical Stimulation of Neuronal Cells by an Organic Semiconductor-Electrolyte Interface. Langmuir 2016, 32, 8533–8542. 10.1021/acs.langmuir.6b02085. 27480642

[ref62] Necas D.; Klapetek P. Gwyddion: an open-source software for SPM data analysis. Cent. Eur. J. Phys. 2012, 10, 181–188.

[ref63] Schneider C. A.; Rasband W. S.; Eliceiri K. W. NIH Image to ImageJ: 25 years of image analysis. Nat. Methods 2012, 9, 671–675. 10.1038/nmeth.2089. 22930834 PMC5554542

[ref64] Balzer F.; Schiek M. In Bottom-Up Self-Organization in Supramolecular Soft Matter; Müller S. C., Parisi J., Eds.; Springer Series in Materials Science; Springer: Berlin, 2015; Chapter 7, Vol. 217, pp 151–176.

[ref65] Rezakhaniha R.; Agianniotis A.; Schrauwen J.; Griffa A.; Sage D.; Bouten C.; van de Vosse F.; Unser M.; Stergiopulos N. Experimental investigation of collagen waviness and orientation in the arterial adventitia using confocal laser scanning microscopy. Biomech. Model. Mechanobiol. 2012, 11, 461–473. 10.1007/s10237-011-0325-z. 21744269

