# Supplementary material for: Template and Temperature-Controlled Polymorph Formation in Squaraine Thin Films
Source: Langmuir. 2022 Jul 20;38(30):9266–77. doi: 10.1021/acs.langmuir.2c01023 (PMC9352357; doi:10.1021/acs.langmuir.2c01023)
Supplement: Supplementary file 1 — la2c01023_si_001.pdf [file la2c01023_si_001.pdf]

Supporting Information
Template and Temperature Controlled Polymorph Formation in Squaraine Thin Films

Frank Balzer,[†] Tobias Breuer,[‡] Gregor Witte,[‡] and Manuela Schiek[*,¶,§]

†*SDU Centre for Photonics Engineering, University of Southern Denmark, Alsion 2, DK-6400 Sønderborg, Denmark*

‡*Department of Physics, Philipps University of Marburg, D-35032 Marburg, Germany*

¶*Institute of Physics, University of Oldenburg, D-26111 Oldenburg, Germany*

§*Center for Surface- and Nanoanalytics, Institute for Physical Chemistry (IPC) & Linz Institute for Organic Solar Cells, Johannes Kepler University, A-4040 Linz, Austria*

E-mail: manuela.schiek@jku.at

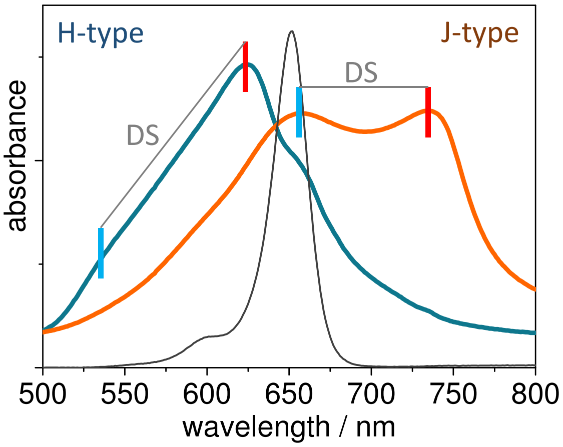

Figure S1: Unpolarized absorbance spectra of SQIB: monomer dissolved in chloroform (grey line), monoclinic polymorph (blue line), and orthorhombic polymorph (orange line).

The monomer absorbance peaks sharply at 650 nm and has a vibronic progression at roughly 600 nm. Both the monoclinic and the orthorhombic polymorph are supported on glass with an out-of-plane alignment with the (0 1 1) and (1 1 0) plane parallel to the substrate. The absorbance spectra in normal incidence transmission show a pronounced projected Davydov splitting (DS) as published earlier in reference 1. The upper (UDC) and lower (LDC) Davydov components are indicated by blue and red bars, respectively. The DS originates from the coupling of translationally inequivalent molecules within the unit cell. One would expect the DS to be centered around the monomer absorbance, resulting in a blue-shifted UDC and a red-shifted LDC, which obviously is not the case here. Instead, both Davydov components are blue-shifted in case of the monoclinic polymorph while they are both red-shifted for the orthorhombic polymorph. This is caused by pronounced coupling between translationally equivalent molecules, that are molecular stacks within the three-dimensional lattice.[2] In case of the monoclinic polymorph this results in an overall H-type aggregate including the H-like vibronic signatures, which is a relative increase of the first vibronic (0-1) progression. The DS splitting energy between the projected (0-1) UDC and LDC transitions indicated in the graph amounts to 0.24 eV. The orthorhombic polymorph can by classified as an overall J-type aggregate with suppressed vibronic progressions. Here, the DS splitting energy amounts to 0.28 eV of the indicated (0-0) UDC and LDC transitions.

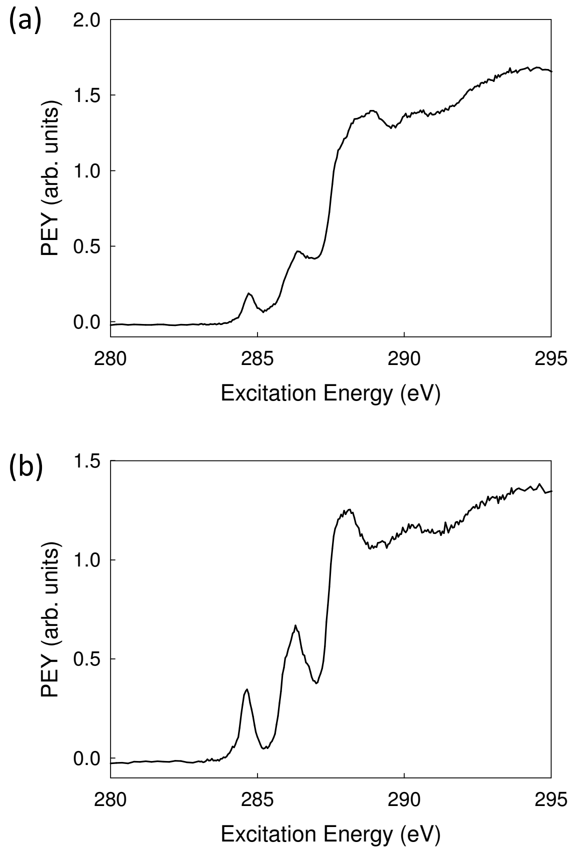

Figure S2: Comparison of C1s-NEXAFS data for an evaporated SQIB film on SiO_2 (Si-wafer covered with native oxide) with nominal thickness of 30 nm (a) and of SQIB powder (b) shows intact sublimation and resublimation of SQIB. No decomposition of the molecules occurs.

NEXAFS data of SQIB powder and a vapor deposited thin film on silicon show the same features, and thus consist of the same molecular compound, documenting that the SQIB molecules are stable during thermal vapor deposition.

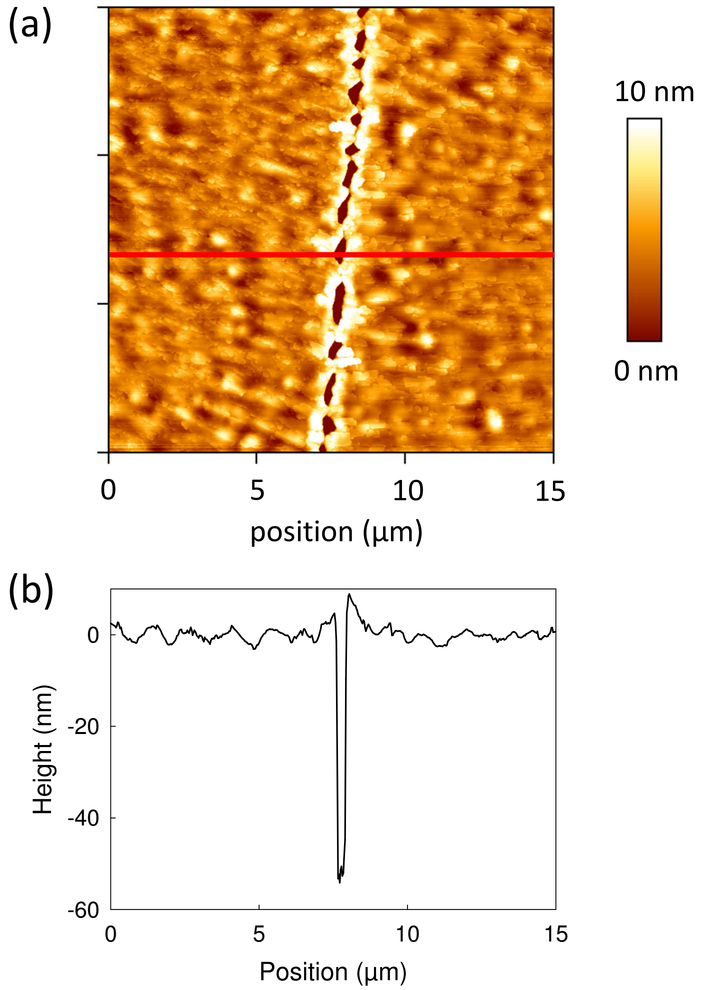

Figure S3: (a) AFM image of a domain boundary between two platelet domains of an annealed SQIB film on glass. The cross section (b) along the red horizontal line in (a) provides the thickness of the film of approximately 50 nm.

Because of the domain size, polarized optical absorption spectroscopy is easiest done for the several 10 µm large orthorhombic platelet domains, Figures S4(a) and (b). The AFM image in Figure S4(a) concurrently displaying both polymorph reveals the substantial roughness of the monoclinic phase due to the lacy texture. This small-sized texture impairs recording absorbance spectra on single monoclinic domains for the postannealed SQIB films on glass.

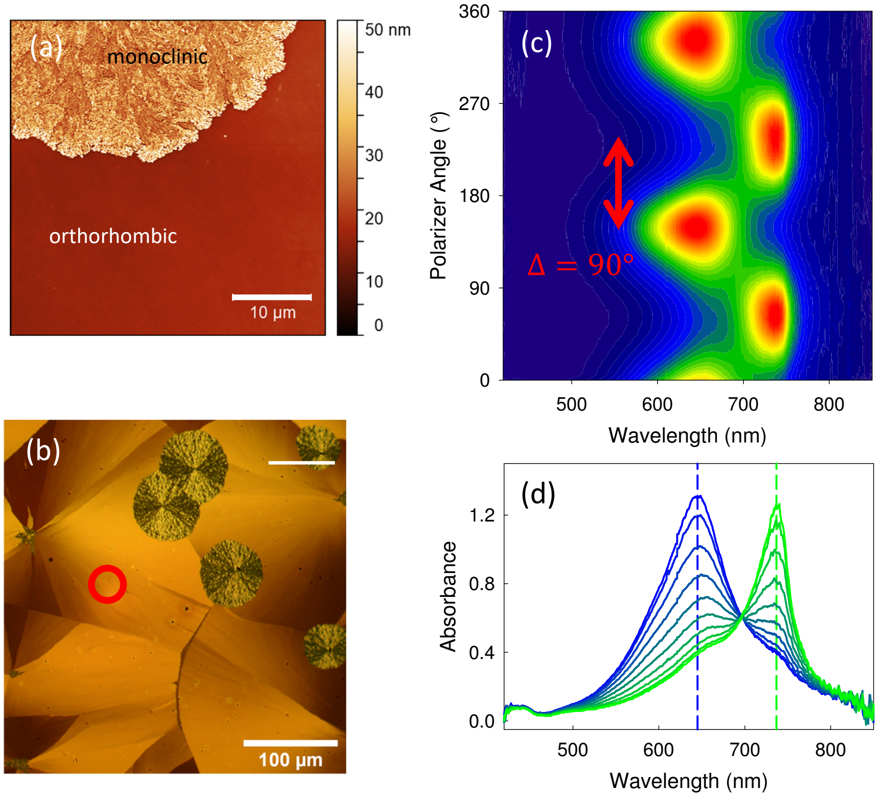

Figure S4: The AFM image (a) elucidates the overall morphology of the two polymorphs of SQIB on glass. In (b), a polarized white light reflection microscopy image is shown. The red circle marks the area, where transmission spectra haven been taken: (c) Polarized absorbance spectra of SQIB platelets. In (d), single spectra are shown where the polarizer is rotated by 10° for every spectrum, covering 90° in total. The different polarization dependence of the upper (UDC) and lower Davydov (LDC) components is obvious. The dashed vertical lines mark the positions of the two maxima.

Even so the extended orthorhombic domains are not single crystalline, parts of them become completely dark when imaged between crossed polarizers.[3,4] In Figures S4(c) and (d), absorbance spectra from part of a single orthorhombic platelet are shown. The position

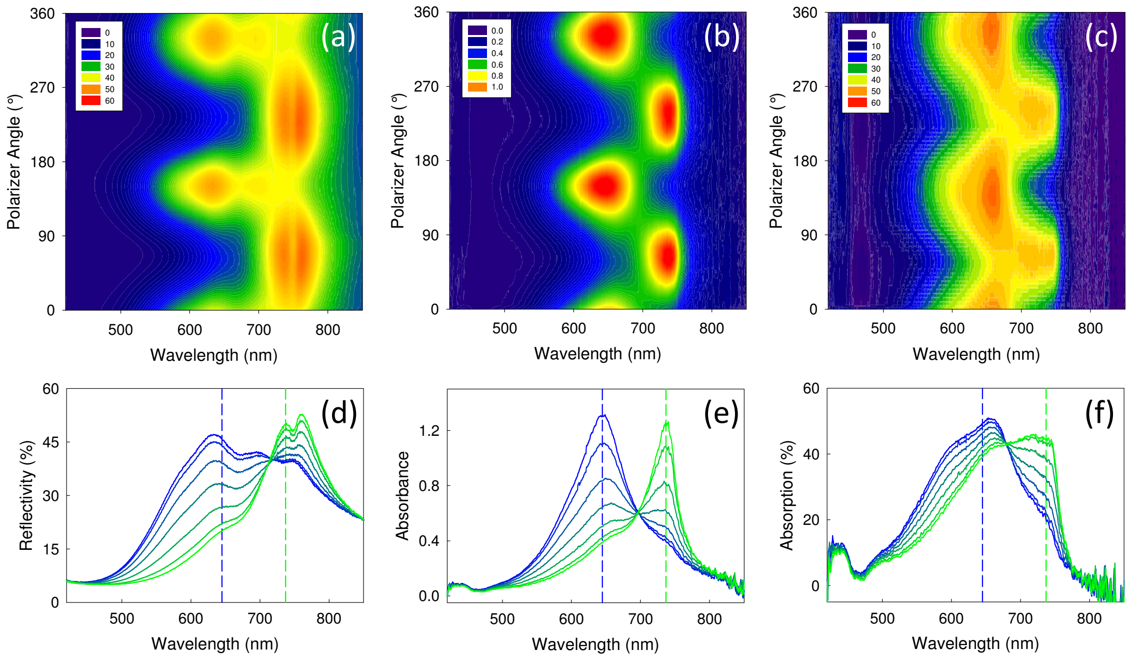

Figure S5: The polarization-resolved specular reflectivity R, (a) and (d), absorbance $Abs = -\log(T)$, (b) and (e), calculated from the transmission T, and from this the calculated absorption $A = 1 - T - R$, (c) and (f), of a single SQIB platelet on glass. Illumination took place from the SQIB side. Vertical dashed lines mark the wavelengths of the two absorbance maxima. Maximal absorbance and reflectivity appear at the same polarisation angle.[3]

where the spectra have been taken is marked in the polarized reflection microscope image (b) by a red circle. Depending on the polarizer direction, either the UDC or LDC dominates the spectrum. As expected for the orthorhombic polymorph with the (1 1 0) plane parallel to the substrate, the angle Δ between the polarizer orientations for the two maxima is 90°. Note that the polarization angles for maximum absorbance also are the angles for maximum reflectivity, Figure S5.

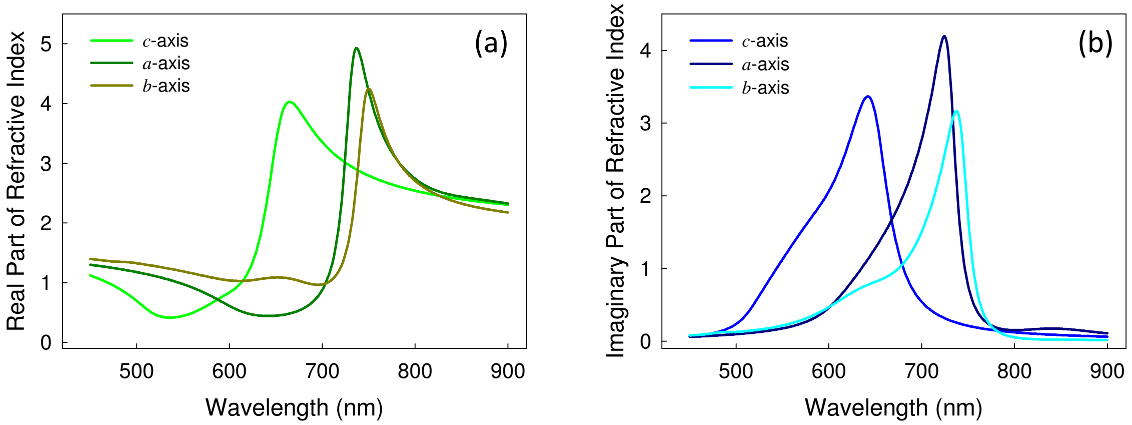

Figure S6: Real (a) and imaginary (b) parts of the complex refractive index $N = n + ik$ along the three crystallographic axes of the orthorhombic SQIB polymorph.[4]

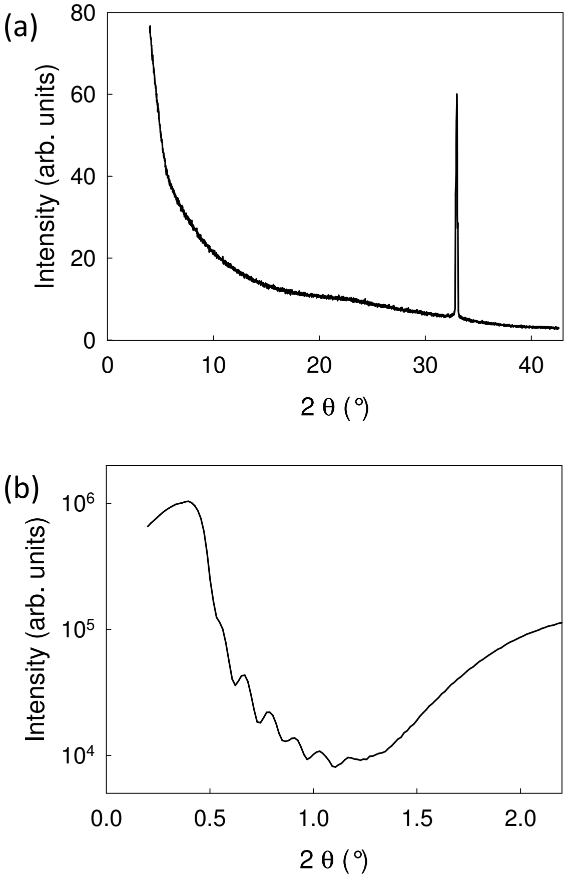

Figure S7: (a) X-ray diffraction of an evaporated SQIB film on SiO_2 (Si-wafer covered with native oxide) with nominal thickness of 30 nm (Cu $K\alpha$ radiation $\lambda = 1.541$ Å). The diffractogram shows no peaks from the SQIB film – the signal at $2\theta \approx 33°$ stems from the Si wafer. (b) Well resolved Kiessig fringes prove the smoothness of the SQIB film.

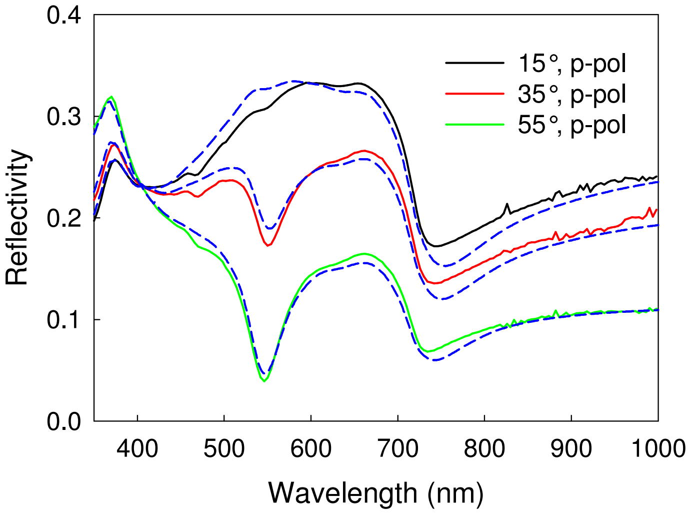

Figure S8: Measured *p*-polarized reflection spectra (solid lines) of an evaporated SQIB film on SiO_2 (Si-wafer covered with 2 nm native oxide) utilizing a J.A. Woollam rotating analyzer ellipsometer (VASE) with vertical sample stage. Dashed lines *p*-polarized reflection spectra based on the complex refractive index presented in Figure 3(b) in the main manuscript. The calculation was performed with OpenFilters setting the thickness of the SQIB layer to 50 nm.[5]

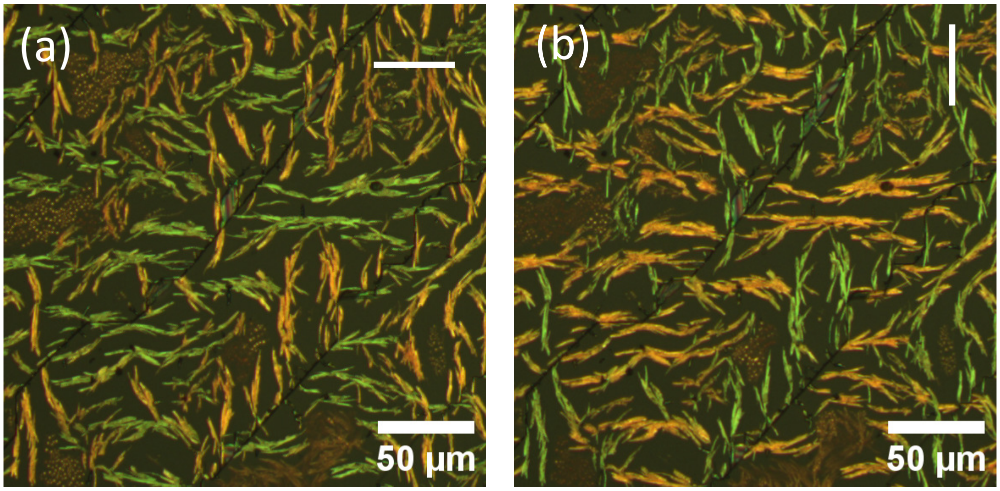

Figure S9: Crossed polarized optical microscopy images of fibrous SQIB crystallites grown by vapor-deposition on KCl for 100 °C substrate temperature. The polarizers are rotated by 90° for (a) and (b). Roughly two main fiber growth directions are noticeable, which suggests an epitaxial alignment of the fibers adopting the monoclinic phase. The fibrillar substructure is too small to be reasonably analyzed further by polarized spectro-microscopy.

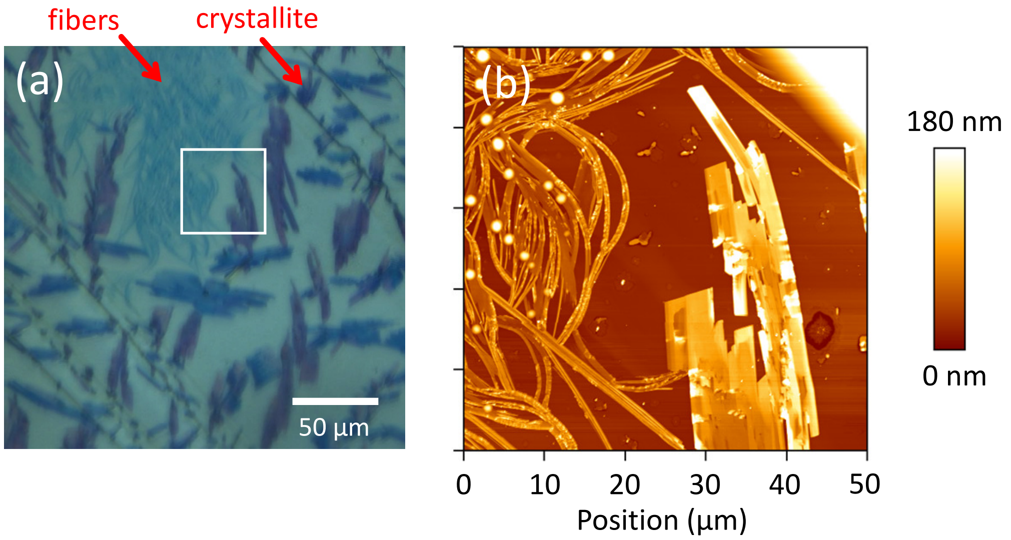

Figure S10: Transmission optical microscope (a) and AFM image (b) of crystallites and fibers grown by vapor-deposition on KCl for 120 °C substrate temperature. The white square in (a) marks the position of the AFM image (b). On top of some fibers, small droplets are often observed. Note, that different colors for the crystallites in (a) stem from a slight linear polarization of the illumination light.

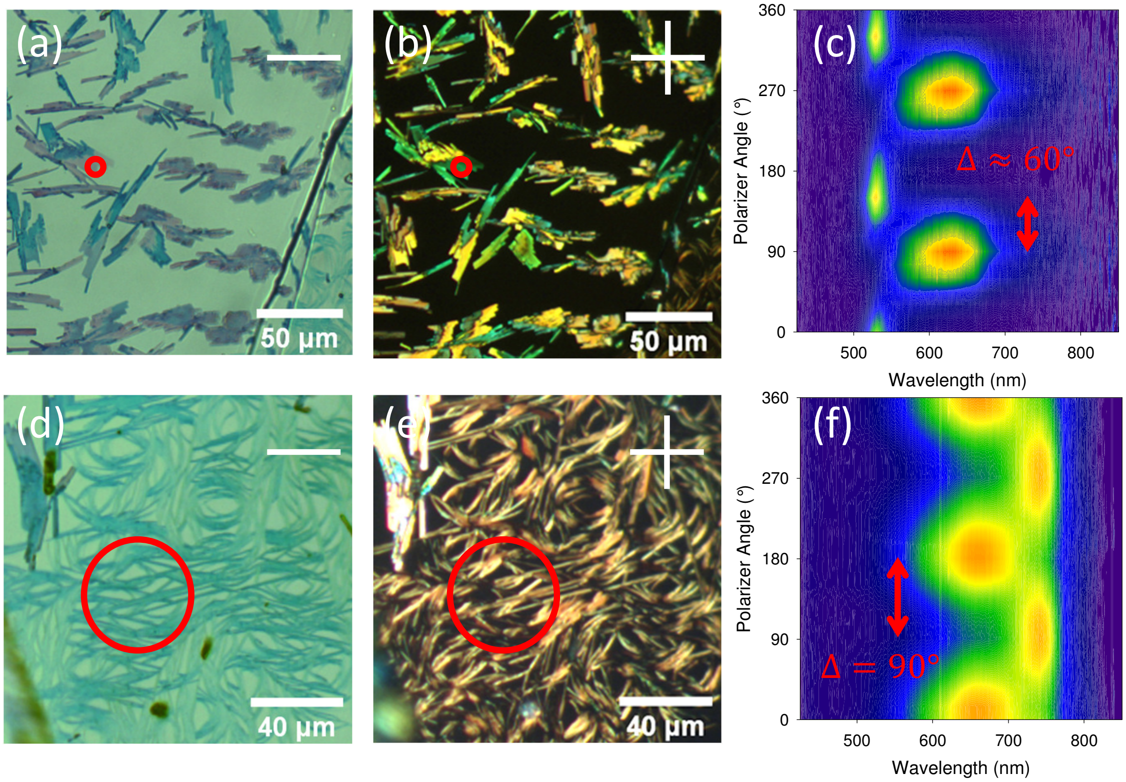

Figure S11: Polarized transmission (single horizontal polarizer) and reflection (crossed polarizers, horizontal and vertical) microscope images of monoclinic phase crystallites (a,b) and orthorhombic phase fibers (d,e) of SQIB formed on KCl(0 0 1) for 120 °C substrate temperature. The corresponding polarized absorbance spectra are shown in (c) and (f), taken at the positions marked by the red circles. The angle Δ provides the angular difference for the absorbance maxima.

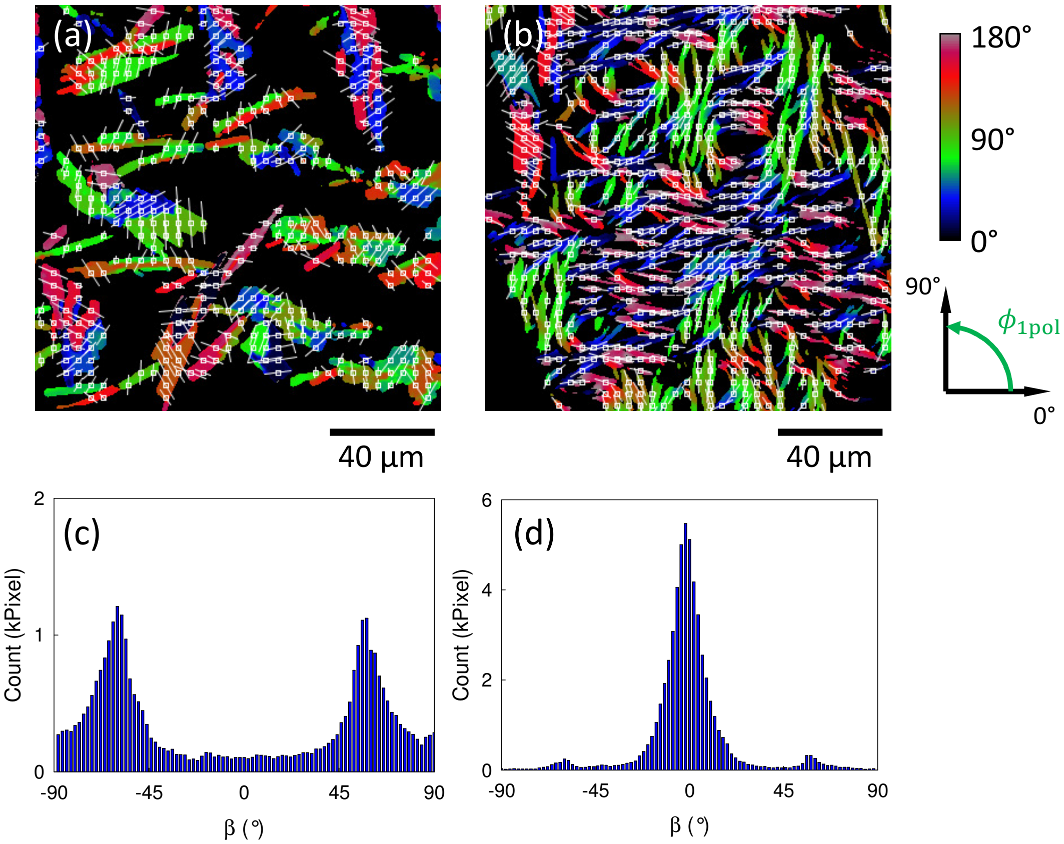

Figure S12: (a) Polarization analysis for the monoclinic phase SQIB crystallites formed on KCl for 120 °C substrate temperature, Figure S11. For this, the wavelength $\lambda = 650\,\mathrm{nm}$ has been chosen, corresponding to the UDC. The white lines together with the color code mark the polarization angle $\phi_{1\mathrm{pol}}$ of maximum reflectivity. (b) The same for the LDC at 650 nm for the orthorhombic phase. In (c) and (d), the angles of maximum reflectivity with respect to the long crystallite direction, $\beta = \phi_{1\mathrm{pol}} - \theta_{\mathrm{orient}}$, are plotted. The angle β is zero if the maximum reflectivity coincides with light polarized along the long crystallite or fiber direction. For the monoclinic phase crystallites, the histogram in (c) shows peaks at $|\beta| = (58 \pm 5)°$. For all of the orthorhombic fibers, $\beta = (0 \pm 5)°$.

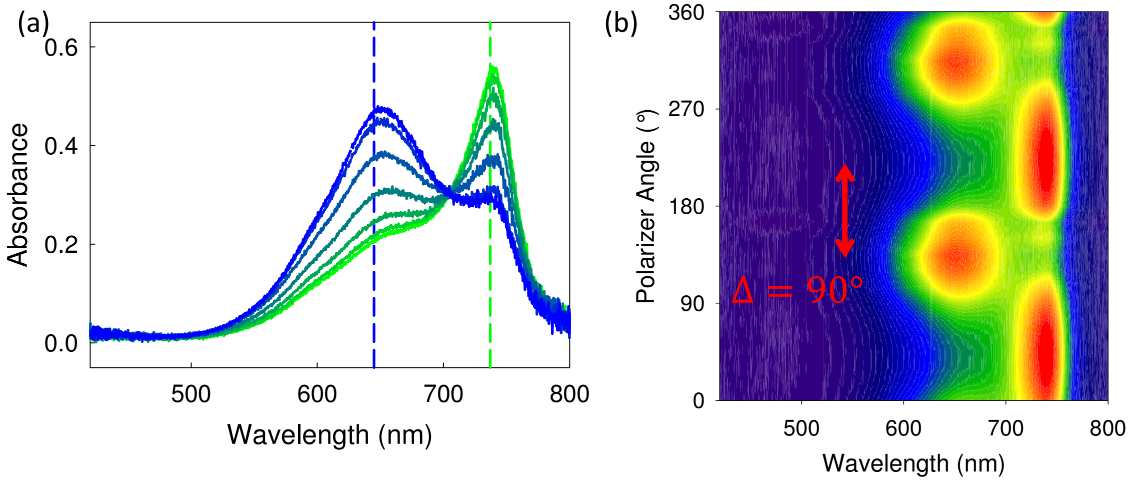

Figure S13: Polarized absorbance spectra of SQIB, vacuum deposited on graphene/quartz. As reference, the bare substrate has been taken for each polarization direction. However, the birefringent quartz substrate leads to a broadening in the polarization angle dependence. The dashed, vertical lines mark the positions of the absorbance maxima for SQIB platelets on glass.

References

(1) Balzer, F.; Kollmann, H.; Schulz, M.; Schnakenburg, G.; Lützen, A.; Schmidtmann, M.; Lienau, C.; Silies, M.; Schiek, M. Spotlight on Excitonic Coupling in Polymorphic and Textured Anilino Squaraine Thin Films. *Cryst. Growth Des.* **2017**, *17*, 6455–6466.

(2) Hestand, N. J.; Spano, F. C. Expanded Theory of H- and J-Molecular Aggregates: The Effect of Vibronic Coupling and Intermolecular Charge Transfer. *Chem. Rev.* **2018**, *118*, 7069–7163.

(3) Balzer, F.; Abdullaeva, O. S.; Maderitsch, A.; Schulz, M.; Lützen, A.; Schiek, M. Nanoscale Polarization-Resolved Surface Photovoltage of a Pleochroic Squaraine Thin Film. *Phys. Status Solidi B* **2020**, *257*, 1900570.

(4) Funke, S.; Duwe, M.; Balzer, F.; Thiesen, P. H.; Hingerl, K.; Schiek, M. Determining the Dielectric Tensor of Microtextured Organic Thin Films by Imaging Mueller Matrix Ellipsometry. *J. Phys. Chem. Lett.* **2021**, *12*, 3053–3058.

(5) Larouche, S.; Martinu, L. OpenFilters: open-source software for the design, optimization, and synthesis of optical filters. *Appl. Opt.* **2008**, *47*, C219–C230.
